# The transcription factor GABPA is a master regulator of naive pluripotency

**DOI:** 10.1038/s41556-024-01554-0

**Published:** 2025-01-02

**Authors:** Chengjie Zhou, Meng Wang, Chunxia Zhang, Yi Zhang

**Affiliations:** 1 Howard Hughes Medical Institute, Boston Children’s Hospital, Boston, MA USA; 2 Program in Cellular and Molecular Medicine, Boston Children’s Hospital, Boston, MA USA; 3 Division of Hematology/Oncology, Department of Pediatrics, Boston Children’s Hospital, Boston, MA USA; 4 Department of Genetics, Harvard Medical School, Boston, MA USA; 5 Harvard Stem Cell Institute, Boston, MA USA; 6 Present Address: State Key Laboratory of Molecular Developmental Biology, Institute of Genetics and Developmental Biology, Chinese Academy of Sciences, Beijing, China

**Keywords:** Pluripotency, Embryonic stem cells, Cell lineage

## Abstract

The establishment of naive pluripotency is a continuous process starting with the generation of inner cell mass (ICM) that then differentiates into epiblast (EPI). Recent studies have revealed key transcription factors (TFs) for ICM formation, but which TFs initiate EPI specification remains unknown. Here, using a targeted rapid protein degradation system, we show that GABPA is not only a regulator of major ZGA, but also a master EPI specifier required for naive pluripotency establishment by regulating 47% of EPI genes during E3.5 to E4.5 transition. Chromatin binding dynamics analysis suggests that GABPA controls EPI formation at least partly by binding to the ICM gene promoters occupied by the pluripotency regulators TFAP2C and SOX2 at E3.5 to establish naive pluripotency at E4.5. Our study not only uncovers GABPA as a master pluripotency regulator, but also supports the notion that mammalian pluripotency establishment requires a dynamic and stepwise multi-TF regulatory network.

## Abstract (web summary)

Zhang and colleagues identify the transcription factor GABPA as a regulator of zygotic genome activation, epiblast formation and naive pluripotency establishment.

## Main

The development of mouse embryos starts with maternal-to-zygotic transition, which is accompanied by maternal RNA degradation and zygotic genome activation (ZGA)^1,2^. After a few rounds of cleavage, the totipotent embryos go through the first cell lineage differentiation to generate inner cell mass (ICM) and trophectoderm (TE) at embryonic day 3.5 (E3.5) (refs. ^3,4^). Subsequently, the ICM cells go through the second cell lineage differentiation to generate epiblast (EPI) and primitive endoderm (PrE) at E4.5 (ref. ^5^).

Transcription factors (TFs) play important roles in cell lineage specification and pluripotency acquisition. At E3.5, the ICM cells express both pluripotency factors such as *Oct4*, *Sox2* and *Nanog* and PrE factors such as *Gata4* and *Gata6*^6^. After completion of the second cell fate specification at E4.5, the expression of lineage-specific TFs becomes restricted with *Gata4* and *Gata6* confined to the PrE, while *Nanog* and *Sox2* are restricted to the EPI, marking the establishment of naive pluripotency in EPI^6^. Given that embryonic stem (ES) cells cannot be fully established from E3.5 ICM^7^, and E3.5 ICM cells have different transcriptome and chromatin accessibility from that of the E4.5 EPI^8^, E3.5 ICM is considered to be at a ‘pre-pluripotency’ state^8^. Recent studies indicate that the TFs NR5A2 and TFAP2C mediate totipotency to pluripotency transition by activating pre-pluripotency genes^9^. However, neither of them is responsible for activating naive pluripotency genes^10^. Moreover, although NANOG and SOX2 are essential for maintaining the naive pluripotency state, they are not required for initiating the naive pluripotency in vivo^11,12^. Thus, the TFs that drive ICM to naive pluripotency transition remain elusive.

Identification of the TFs regulating pluripotency establishment in vivo is hindered by their potential roles in earlier developmental stages. Thus, conventional TF-knockout (KO) mouse models could not separate their potential roles in regulating totipotency or ICM formation from that in regulating EPI formation. The development of a targeted protein degradation system such as auxin-inducible degron (AID)^13^ and degradation tag (dTAG)^14,15^ enables the rapid degradation of target proteins at a specific time window, making the study of gene function at a specific developmental stage possible. In this study, by generating dTAG mice and combining with RNA sequencing (RNA-seq) and low-input CUT&RUN assay, we identified and demonstrated that the TF GA repeat binding protein alpha (GABPA), encoded by the minor ZGA gene *Gabpa*, plays an essential role in naive pluripotency establishment in vivo. Our results revealed that GABPA is not only important for major ZGA, but also critical for ICM to naive pluripotency transition by regulating a large set of pluripotency genes through binding to their promoters. Importantly, we found that, during ICM-to-EPI transition, the decreased binding of TFAP2C and SOX2 at the promoters of certain ICM genes was concomitant with the increased binding of GABPA at these gene promoters, indicating a switch in the key TFs that regulate pluripotency gene expression. These results support a dynamic and stepwise regulatory model for naive pluripotency establishment during pre-implantation development.

## Results

### Identification of GABPA as a potential pluripotency regulator

**Fig. 1:**
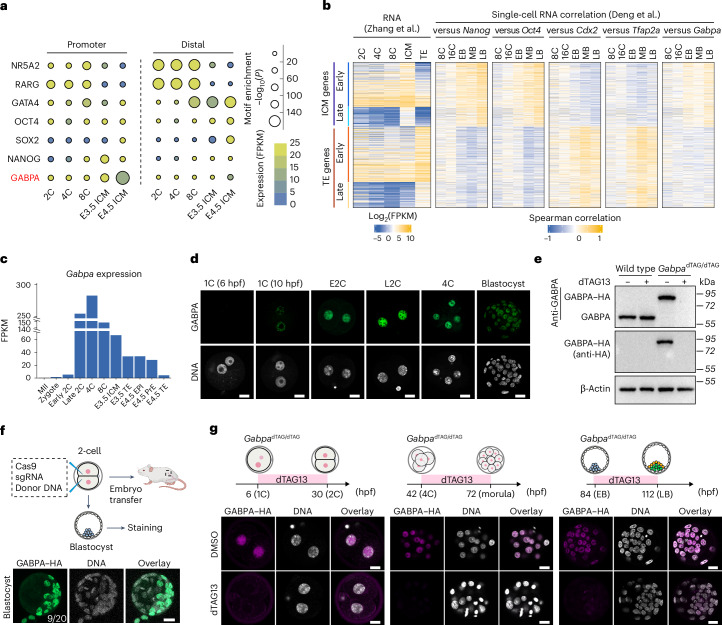
Identification of *Gabpa* as a potential pluripotency regulator and generation the *Gabpa*^dTAG/dTAG^ mice. **a**, Enrichment of TF motifs at promoter (left) and distal (right) ATAC–seq peaks (two biological replicates of each developmental stage were merged in this analysis) during mouse pre-implantation development. **b**, Expression correlation of ICM genes and TE genes with the expression of *Gabpa* and other known ICM markers (*Nanog* and *Oct4*) and TE markers (*Cdx2* and *Tfap2a*) at the 8-cell (8C), 16-cell (16C), early blastocyst (EB), middle blastocyst (MB) and late blastocyst (LB) stages at the single-cell level. Early ICM/TE genes are genes with eight-cell expression (FPKM ≥1); late ICM/TE genes are genes starting expression at E3.5 with no eight-cell expression (FPKM <1). Two biological replicates were merged. **c**, *Gabpa* expression levels during mouse pre-implantation development. **d**, Immunostaining of GABPA (green) during mouse pre-implantation development. DNA, Hoechst 33342. Scale bar, 20 μm. **e**, A western blot confirming GABPA degradation with dTAG13 in ES cells. The blots were incubated with anti-GABPA and anti-HA, respectively. β-Actin was used as a loading control. **f**, The strategy used for *Gabpa*^dTAG/dTAG^ mouse generation. HA staining indicates the dTAG knock-in cells in blastocyst. DNA, Hoechst 33342. Scale bar, 20 μm. **g**, Immunostaining confirming GABPA degradation with dTAG13 treatment at different pre-implantation developmental stages. Anti-HA antibody was used for endogenous GABPA–HA fusion protein staining. DNA, Hoechst 33342. Scale bar, 20 μm. Experiments in **d**–**g** were repeated three independent times based on independent biological samples. 1C, one-cell; E2C, early two-cell; L2C, late two-cell. Source data

To identify candidate TFs potentially involved in ICM to naive EPI differentiation, we performed integrative analyses of public RNA-seq and assay for transposase-accessible chromatin with sequencing (ATAC–seq) datasets from mouse pre-implantation embryos^16–19^. Compared with OCT4, NANOG and SOX2 binding motif, GABPA binding motif is highly enriched in the open promoters of E4.5 ICM (EPI + PrE), suggesting GABPA may have a role in regulating E4.5 ICM formation (Fig. 1a). Consistent with a previous study^16^, GABPA binding motif is not enriched in distal open chromatin (Fig. 1a). In addition, single-cell gene expression correlation analysis between various TFs and the ICM genes or TE genes (Supplementary Table 1) revealed that *Gabpa* expression positively correlates with expression of ICM genes in blastocysts, particularly at late blastocyst stage similar to that of *Oct4* and *Nanog* (Fig. 1b), indicating potential involvement of *Gabpa* in pluripotency regulation. Furthermore, *Gabpa* transcription starts from the zygote to the early two-cell stage, reaches its highest level at late two-cell and four-cell stages, and then becomes differentially expressed in ICM and TE at the blastocyst stage (Fig. 1c). Immunostaining indicated that GABPA can be detected from zygote to blastocyst (Fig. 1d). These data are consistent with a potential role of GABPA in regulating pluripotency.

**Extended Data Fig. 1:**
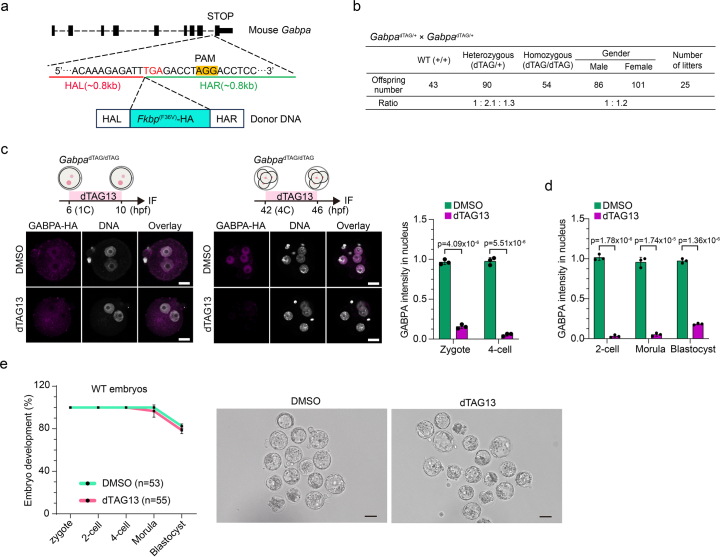
Generation of *Gabpa*^dTAG/dTAG^ mice. (**a**) Diagram of *Fkbp*^(F36V)^-HA knock-in at the 3’ end of *Gabpa* gene. (**b**) Genotypes of pups from heterozygous *Gabpa*^dTAG/+^× *Gabpa*^dTAG/+^ crosses. (**c**) Confirmation of GABPA degradation with 4h-dTAG13 treatment in zygote (left) and 4-cell (middle), and anti-HA fluorescence intensity quantification (right) (n = 3). Anti-HA antibody was used for GABPA-HA fusion protein staining. DNA, Hoechst 33342. Scale bar, 20 μm. (**d**) Quantification of the fluorescence intensity of GABPA in Fig. 1g (n = 3). (**e**) Embryo development of wild-type embryos with or without dTAG 13 treatment. The developmental rate (left) and representative pictures of E3.5 embryos (middle and right). Scale bar, 50 μm. The independent experiments were repeated three times. Data in c d and e are presented as mean values ± SD. P-values were calculated with two-sided Student’s t-test.

GABPA is a member of the erythroblast transformation specific (ETS) TF family, which forms a tetrameric complex with GABPB to regulate the transcriptional activation^20^. Previous studies have shown that *Gabpa* KO resulted in embryonic lethality before the blastocyst stage^21^, but its role in cell fate specification is unknown. To assess its role in ICM and EPI specification, we utilized the dTAG system^14^. Given the potential instability of the dTAG fusion protein^22^, we first tested the GABPA dTAG system in mouse ES cells, which showed that GABPA–FKBP (FK506 binding proteins)–HA (hemagglutinin) and the endogenous GABPA proteins are at similar levels (Fig. 1e and Extended Data Fig. 1a), indicating that the FKBP–HA fusion does not affect GABPA stability. Importantly, dTAG13 treatment resulted in complete GABPA–FKBP–HA degradation within 30 min (Fig. 1e). We thus proceeded to generate the mouse harbouring the *Gabpa*^dTAG^ allele using the CRISPR technology (Fig. 1f and Extended Data Fig. 1a,b)^23^. Importantly, addition of dTAG13 to the cultured embryos can efficiently degrade GABPA at zygote, two-cell, four-cell, morula and blastocyst stages in both short- and long-time-window treatments (Fig. 1g and Extended Data Fig. 1c,d). Moreover, dTAG13 treatment did not affect the development of wide-type embryos (Extended Data Fig. 1e). Collectively, these results demonstrate the successful generation of a GABPA-dTAG mouse model.

### GABPA activates a group of major ZGA genes by binding to their promoters

**Fig. 2:**
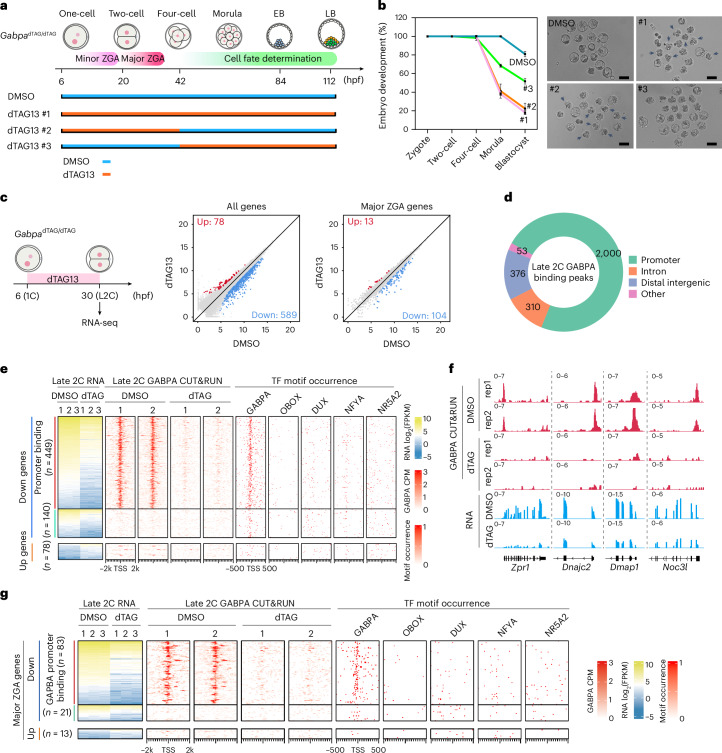
GABPA activates a group of major ZGA genes by binding to their promoters. **a**, A diagram showing dTAG13-triggered GABPA degradation at different time windows of mouse pre-implantation development. dTAG13 #1: dTAG13 treatment from zygote (6 hpf) to late blastocyst (112 hpf) stage; dTAG13 #2: dTAG13 treatment from zygote to four-cell (42 hpf) stage (covers ZGA stage). dTAG13 #3: dTAG13 treatment from four-cell to late blastocyst stage (after ZGA). **b**, The embryo development rate (left) and representative images of blastocyst stage embryos (right) after GABPA degradation (DMSO, *n* = 43; #1, *n* = 50; #2, *n* = 57; #3, *n* = 54; *n* represents the total embryos from three independent experiments). The data are presented as mean values ± standard deviation. The black arrows indicate the four-cell or eight-cell arrested embryos. Scale bar, 100 μm. **c**, An RNA-seq comparison of late two-cell embryos treated with dTAG13 or DMSO from zygote (6 hpf) to late two-cell stage (30 hpf). The *x* and *y* axis of the dot plots are log_2_-normalized counts from RNA-seq. **d**, The genomic distribution of GABPA binding peaks generated by GABPA CUT&RUN at late two-cell stage. **e**, Heatmaps showing the DEGs at late two-cell stage upon GABPA degradation, and the GABPA binding, TF motif occurrence around the TSS of corresponding genes. The downregulated genes were separated into two groups based on whether they have direct GABPA binding. **f**, Genome browser examples of GABPA target genes at late two-cell stage. **g**, Heatmaps showing the differentially expressed major ZGA genes and GABPA binding profile in late two-cell embryos after GABPA degradation. TF motif occurrences around the TSS of corresponding ZGA genes are shown. The downregulated genes were separated into two groups based on whether they have direct GABPA binding. Three biological replicates were used for RNA-seq analysis in **c**, **e** and **g**. The RNA-seq examples in **f** are shown as the pooled results of three biological replicates in each condition. Two biological replicates were used for CUT&RUN analysis in **d**–**g**. 1C, one-cell; L2C, late two-cell; EB, early blastocyst; LB, late blastocyst. Source data

To understand when and how GABPA degradation affects embryonic development, we performed dTAG13 treatment at different time windows. Given that *Gabpa* starts to express after fertilization (Fig. 1c), we confirmed that GABPA is a minor ZGA gene with a detectable protein level at 10 h post fertilization (hpf) (Fig. 1d). We therefore performed GABPA degradation starting at 6 hpf with continuous dTAG13 presence (Fig. 2a, #1), which caused most embryos to arrest at the four-cell or eight-cell stage (Fig. 2b, #1, arrows), suggesting GABPA plays important roles before the four-cell stage. Since GABPA is highly expressed at late two-cell stage (Fig. 1c,d), we asked whether GABPA regulates major ZGA. To this end, we treated the embryos with dTAG13 from 6 to 42 hpf and then washed out dTAG13 (Fig. 2a,b, #2). This treatment affected embryo development similarly to when dTAG13 is continuously present from 6 to 112 hpf. Moreover, GABPA degradation after major ZGA (42–112 hpf) resulted in a much weaker phenotype (Fig. 2a,b, #3), suggesting that GABPA plays an important role in ZGA.

**Extended Data Fig. 2:**
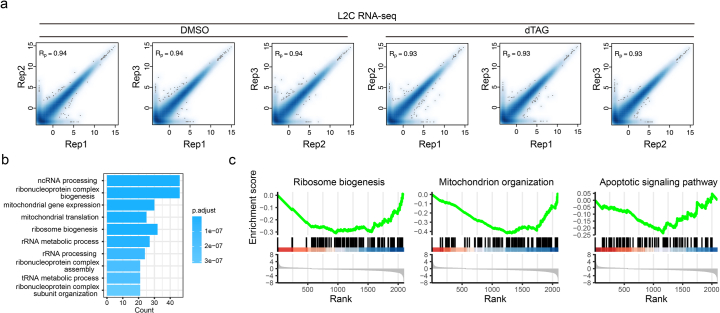
Genes affected by GABPA degradation at late 2-cell. (**a**) Correlations of the replicates of the RNA-seq at late 2-cell stage. R_p_: Pearson correlation. (**b**) Gene Ontology (GO) terms enriched for all the down-regulated genes after dTAG13 treatment at late 2-cell stage. P-values were adjusted with Benjamini-Hochberg method. (**c**) Gene set enrichment analysis (GSEA) of all the down-regulated genes at late 2-cell stage after dTAG13 treatment for ribosome biogenesis (left), mitochondrion organization (middle) and apoptotic signaling pathway (right).

To study GABPA’s role in regulating major ZGA, we treated the *Gabpa*^dTAG/dTAG^ embryos with dTAG13 from mid-zygote (6 hpf) to late two-cell stage (30 hpf) and collected embryos at 30 hpf for RNA-seq (Fig. 2c and Extended Data Fig. 2a), which revealed 589 downregulated and 78 upregulated genes (Fig. 2c). Gene Ontology (GO) analysis revealed that the downregulated genes are involved in processes such as ribosome biogenesis, ribosomal RNA processing and so on (Extended Data Fig. 2b,c), explaining the embryo arrest phenotype. In contrast, the upregulated genes did not show GO term enrichment. Further analysis of the downregulated genes identified 104 major ZGA genes, indicating that GABPA has a role in activating these major ZGA genes (Fig. 2c and Supplementary Tables 1 and 2).

**Extended Data Fig. 3:**
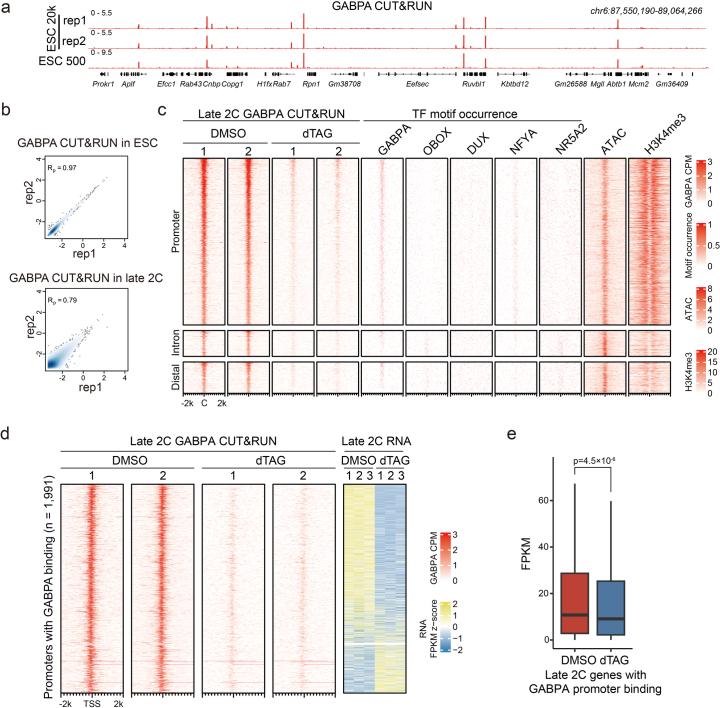
GABPA binding profiles at the late 2-cell stage. (**a**) Genome browser view showing examples of GABPA CUT&RUN profiles in ESCs using 500 (n = 1 independent biological replicate) and 20k cells (n = 2 independent biological replicates). (**b**) Correlation of replicates for GABPA CUT&RUN with ESCs (top) and late 2-cell embryos (bottom). (**c**) Heatmaps showing the GABPA binding profiles at promoter, intron and distal regions in late 2-cell embryos. TFs motif occurrence, ATAC and H3K4me3 signals are shown at the corresponding regions. C: peak center. (**d**) Heatmaps showing GABPA promoter binding at late 2-cell stage and the corresponding genes expression level with or without the dTAG13 treatment. (**e**) Boxplot comparing the expression levels of GABPA targeted genes in DMSO and dTAG-13 treated late 2-cell (For late 2C genes with GABPA promoter binding, n = 1,991). P-values were calculated with two-sided Mann-Whitney U test. In the boxplot, the central band represents the median. The lower and upper edges of the box represent the first and third quartiles, respectively. The whiskers of the boxplot extend to 1.5 times interquartile range (IQR). GABPA CUT&RUN analysis in **c** and **d** were performed using two independent biological replicates. ATAC-seq and H3K4me3 CUT&RUN in **c** showed the pooled results from two biological replicates.

To determine whether GABPA directly binds to and regulates the downregulated genes, we performed low-input CUT&RUN on GABPA^24,25^. We first tested GABPA CUT&RUN in mouse ES cells and found the use of 500 ES cells generated similar results as when using 20,000 cells (Extended Data Fig. 3a,b). We then performed GABPA CUT&RUN using late two-cell embryos (Extended Data Fig. 3b). Analysis of the CUT&RUN data indicated that most GABPA binding peaks are located in the nucleosome-depleted regions of promoters (Fig. 2d and Extended Data Fig. 3c). Interestingly, the GABPA binding regions were enriched for the GABPA motif but not the motifs of other murine ZGA regulators such as OBOX, DUX or NR5A2^26–28^, suggesting direct GABPA binding at these regions (Extended Data Fig. 3c). A clear GABPA binding signal around the transcription start sites (TSSs) of downregulated genes (449 out of 589), but not upregulated genes, was observed (Fig. 2e,f). Importantly, most (83 out of 104) downregulated major ZGA genes also showed direct GABPA promoter binding (Fig. 2f,g). The fact that GABPA degradation caused loss of promoter binding, resulting in the downregulation of most GABPA direct targets (Extended Data Fig. 3d,e), including some major ZGA genes (Fig. 2c,g), supports that *Gabpa* plays an important role in activating these major ZGA genes by direct promoter binding.

### GABPA controls EPI specification by activating pluripotency genes

**Fig. 3:**
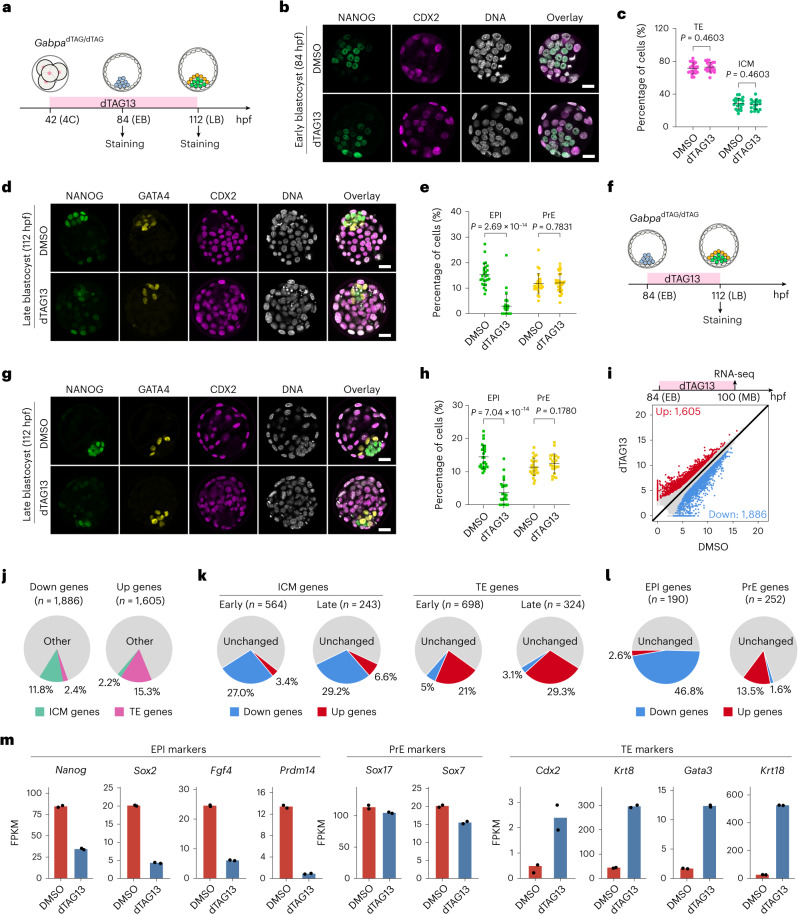
GABPA controls EPI specification by activating pluripotency genes. **a**, A diagram showing the dTAG13 treatment after major ZGA and sample collection at early (84 hpf) and late (112 hpf) blastocyst stages. **b**, Immunostaining of NANOG (green) and CDX2 (red) at early blastocyst stage with or without dTAG13 treatment. Scale bar, 20 μm. **c**, The percentage of ICM and TE cells quantified on the basis of **b** (DMSO, *n* = 21; dTAG13, *n* = 19; *n* represents total embryos of three independent experiments). ICM: NANOG^+^/CDX2^−^. TE: CDX2^+^. *P* values were calculated with Student’s *t*-test (two-sided). The data are presented as mean values ± standard deviation (s.d.). **d**, Immunostaining of NANOG (green), GATA4 (yellow) and CDX2 (red) at middle blastocyst stage with or without dTAG13 treatment. Scale bar, 20 μm. **e**, The percentage of EPI and PrE cells quantified on the basis of **d** (DMSO, *n* = 26; dTAG13, *n* = 30; *n* represent total embryos of four independent experiments). EPI: NANOG^+^/GATA4^−^/CDX2^−^. PrE: GATA4^+^/NANOG^−^/CDX2^−^. *P* values were calculated with Student’s *t*-test (two-sided). The data are presented as mean values ± s.d. **f**, A diagram showing dTAG13 treatment from early to late blastocyst stage. **g**, The same as **d** except the dTAG13 treatment time is as indicated in **f**. Scale bar, 20 μm. **h**, The percentage of EPI and PrE cells quantified on the basis of **g** (DMSO, *n* = 26; dTAG13, *n* = 24; *n* represent total embryos of three independent experiments). EPI: NANOG^+^/GATA4^−^/CDX2^−^. PrE: GATA4^+^/ NANOG^−^/CDX2^−^. *P* values were calculated with Student’s *t*-test (two-sided). The data are presented as mean values ± s.d. **i**, DEGs from RNA-seq of E4.5 (late blastocyst) ICM with or without dTAG13 treatment. **j**, The percentage of differentially expressed ICM and TE genes. **k**, The percentage of affected early/late ICM and TE genes after dTAG13 treatment. **l**, The percentage of up- and downregulated EPI and PrE genes after dTAG13 treatment. **m**, Expression levels of EPI, PrE and TE marker genes with or without dTAG13 treatment. 4C, four-cell; EB, early blastocyst; MB, middle blastocyst; LB, late blastocyst.

**Extended Data Fig. 4:**
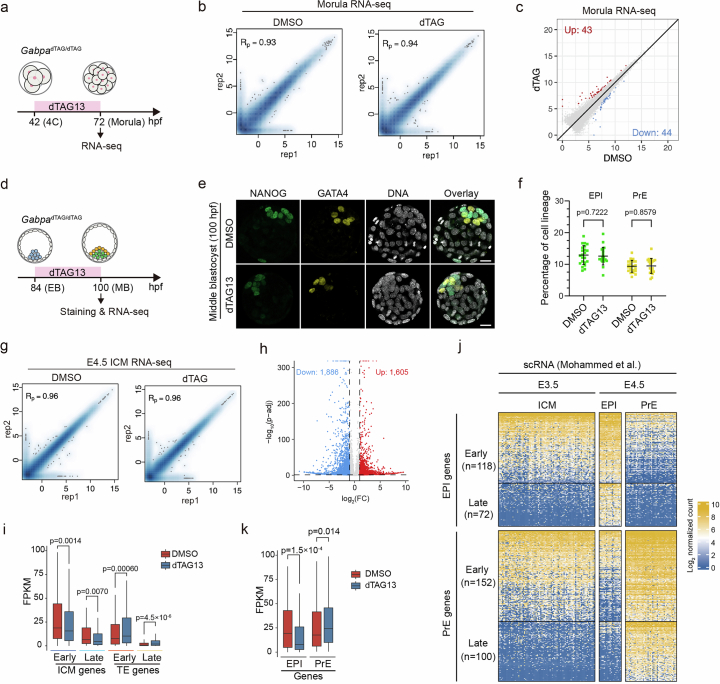
RNA-seq in morula and E4.5 ICM in response to dTAG13 treatment after major ZGA. (**a**) Diagram showing dTAG13 treatment time window and the time of sample collection for RNA-seq. (**b**) Correlation of RNA-seq replicates at morula stage. R_p_: Pearson correlation. (**c**) Dot plot showing the DEGs in morula after dTAG13 treatment shown in panel a. (**d**) Diagram showing the times of dTAG13 treatment and sample collection. EB: early blastocyst, MB: middle blastocyst. (**e**) Immunostaining of NANOG (green) and GATA4 (yellow) for middle blastocyst in control (DMSO) and GABPA degradation (dTAG13) conditions. Scale bar, 20 μm. (**f**) Percentage of EPI and PrE cells of middle blastocyst in control (DMSO, n = 22) and GABPA degradation (dTAG13, n = 21) conditions. n indicates the total embryo numbers used in three independent experiments. P-values were calculated with two-sided Student’s t-test. Data are presented as mean values ± SD. (**g**) Correlation of replicates of RNA-seq in E4.5 ICM. (**h**) Volcano plot showing the DEGs after GABPA degradation in E4.5 ICM. P-values were adjusted with Benjamini-Hochberg method. (**i**) Boxplot comparing the expression levels of early ICM genes (n = 564), late ICM genes (n = 243) and early TE genes (n = 698), late TE genes (n = 324) at E4.5 ICM with or without dTAG13 treatment. P-values were calculated with two-sided Mann-Whitney U test. In the boxplot, the central band represents the median. The lower and upper edges of the box represent the first and third quartiles, respectively. The whiskers of the boxplot extend to 1.5 times interquartile range (IQR). (**j**) EPI genes and PrE genes identified from single-cell RNA-seq data from Mohammed et al. 99 cells from 10 E3.5 ICM and 105 cells from 5 E4.5 ICM were used for analysis. (**k**) Boxplot comparing the expression levels of EPI genes (n = 190) and PrE genes (n = 252) at E4.5 ICM with or without dTAG13 treatment. P-values were calculated with two-sided Mann-Whitney U test. In the boxplot, the central band represents the median. The lower and upper edges of the box represent the first and third quartiles, respectively. The whiskers of the boxplot extend to 1.5 times interquartile range (IQR).

Next, we asked whether GABPA has a role in the first cell lineage specification. To avoid ZGA defect, we treated *Gabpa*^dTAG/dTAG^ embryos with dTAG13 starting from the four-cell stage and collected early (84 hpf) and late (112 hpf) blastocysts for immunostaining (Fig. 3a). Neither ICM nor TE cell numbers in early blastocysts were affected by the treatment (Fig. 3b,c), suggesting GABPA is not required for the first cell lineage specification. However, for late blastocyst stage, the EPI, but not the PrE, cells were dramatically decreased by the treatment (Fig. 3d,e). RNA-seq analysis of morula embryos revealed a limited transcriptome change upon the treatment (Extended Data Fig. 4a–c and Supplementary Table 3), consistent with minor role of GABPA from four-cell to morula embryos. To avoid a potential contribution from defects between the four-cell stage and early blastocyst stage, we started the treatment in early blastocyst stage when the first cell lineage specification has already finished and then analysed the effect on EPI and PrE at late blastocyst stage (Fig. 3f). A similar effect of this treatment to that of the treatment started at four-cell embryos was observed (Fig. 3g,h), indicating that GABPA has a direct role in EPI specification.

To understand how GABPA regulates EPI specification, we performed RNA-seq. To capture the earlier molecular changes that lead to EPI defects, and to avoid potential confounding due to EPI and PrE cell ratio change, we collected ICM cells at the mid-blastocyst stage (100 hpf) when the EPI cell number had not yet shown a difference between dimethyl sulfoxide (DMSO) and dTAG13 treatment (Extended Data Fig. 4d–f). Transcriptome analyses revealed 1,605 up- and 1,886 downregulated genes in response to GABPA degradation (Fig. 3i, Extended Data Fig. 4g,h and Supplementary Table 4). Some pluripotency factors have already expressed at E3.5 ICM when GABPA degradation starts (Extended Data Fig. 4d); although EPI could form at middle blastocyst, these EPI cells are defective in their transcriptome due to GABPA degradation (Fig. 3i). Importantly, 223 of the 1,886 (11.8%) downregulated genes belong to the ICM genes, while 246 of the 1,605 (15.3%) upregulated genes belong to the TE genes (Fig. 3j). Further analysis revealed that 27% of the early ICM genes and 29.2% of the late ICM genes were downregulated, while 21% of the early TE genes and 29.3% of the late TE genes were upregulated by GABPA degradation, indicating that GABPA plays an essential role in pluripotency establishment (Fig. 3k and Extended Data Fig. 4i).

We further found 46.8% of EPI genes were downregulated (Fig. 3l,m, Extended Data Fig. 4j and Supplementary Table 1). In contrast, a much smaller percentage of PrE genes (1.6%) were downregulated, and importantly, most PrE marker genes, such as *Sox17* and *Sox7*, did not show significant change (Fig. 3l,m and Extended Data Fig. 4k), consistent with the observation that EPI, but not PrE, was affected upon GABPA degradation (Fig. 3d–h). Collectively, our data support that GABPA determines EPI but not PrE specification by regulating EPI genes.

### GABPA regulates EPI gene expression by promoter and distal binding

**Fig. 4:**
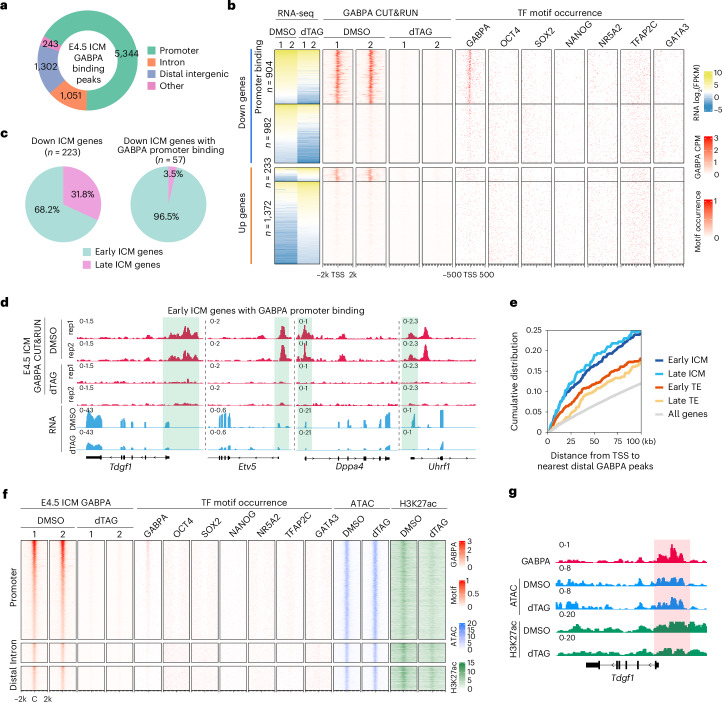
GABPA regulates EPI genes in E4.5 ICM by both promoter and distal binding. **a**, The genomic distribution of GABPA binding peaks in E4.5 ICM based on CUT&RUN data. **b**, Heatmaps showing the DEGs at E4.5 ICM after dTAG13 treatment, as well as the GABPA binding and several TF motif occurrences around the TSS of the indicated gene groups. **c**, The percentage of the downregulated early and late ICM genes (left), and downregulated ICM genes with GABPA promoter binding (right) at E4.5 ICM. **d**, Examples of early ICM genes with GABPA promoter binding (shaded) at E4.5 ICM. **e**, Cumulative distributions of the distance from TSS to the nearest distal GABPA peaks for each group of genes at E4.5 ICM. **f**, Heatmaps showing all the GABPA peaks at promoters, introns and distal regions in E4.5 ICM, and ATAC–seq and H3K27ac signals at these GABPA peak regions with or without dTAG13 treatment. C: peak centre. **g**, An example of the genome browser view of GABPA binding (shaded), ATAC and H3K27ac signals in E4.5 ICM with or without dTAG13 treatment. All RNA-seq, GABPA CUT&RUN, ATAC–seq and H3K27ac CUT&RUN analysis were performed using two independent biological replicates. The pooled results for ATAC–seq and H3K27ac CUT&RUN are shown in **f** and **g**.

**Extended Data Fig. 5:**
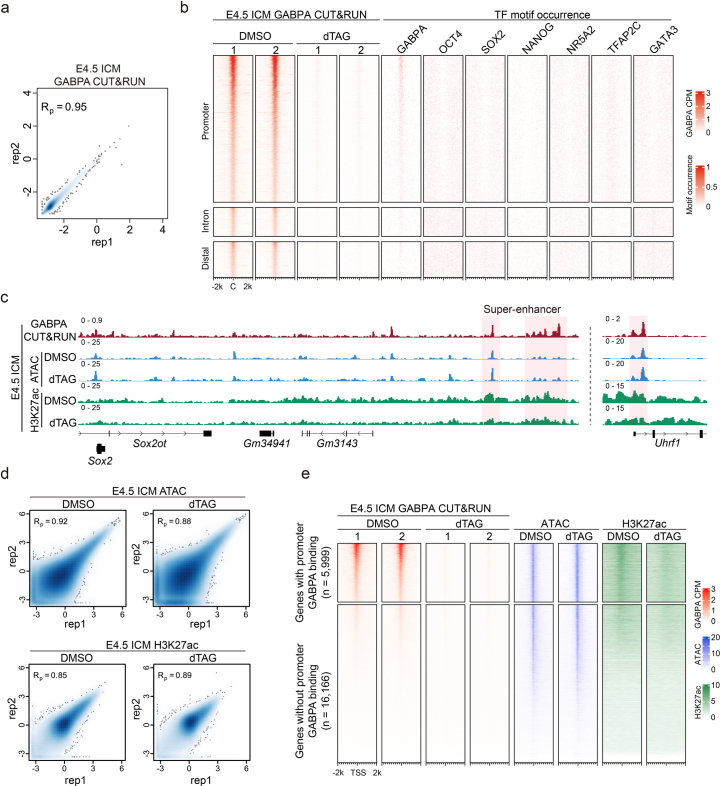
GABPA CUT&RUN in E4.5 ICM. (**a**) Correlation of replicates for GABPA CUT&RUN in E4.5 ICM. R_p_: Pearson correlation. (**b**) Heatmaps showing the GABPA binding profiles at promoter, intron and distal regions in E4.5 ICM, and the TFs motif occurrence at the corresponding regions (n = 2 independent biological replicates). C: peak center. (**c**) Genome browser view of examples of GABPA binding, ATAC and H3K27ac signals at E4.5 ICM. The binding sites are shaded. (**d**) Correlation of replicates for ATAC-seq and H3K27ac CUT&RUN with or without dTAG13 treatment in E4.5 ICM. R_p_: Pearson correlation. (**e**) Heatmap showing GABPA binding (n = 2 independent biological replicates), ATAC-seq and H3K27ac signals at gene promoters in E4.5 ICM. The genes were separated into two groups based on whether they have promoter GABPA binding. For ATAC and H3K27ac, two replicates in each condition were merged. GABPA CUT&RUN in **c**, ATAC-seq and H3K27ac CUT&RUN in **c** and **e** showed the pooled results from two biological replicates.

To understand how GABPA regulates the ICM genes in E4.5 embryos, we performed GABPA CUT&RUN using E4.5 ICM cells (Extended Data Fig. 5a), which revealed that GABPA mainly occupies the promoter regions (Fig. 4a and Extended Data Fig. 5b). Motif analysis revealed that these regions are enriched for the GABPA motif, but not motifs of other lineage regulators including OCT4, SOX2, NANOG and so on, indicating GABPA plays a direct role by binding to these regions (Extended Data Fig. 5b). Integrative analyses of the GABPA CUT&RUN and RNA-seq data revealed that almost half of the downregulated genes (904 out of 1,886) were bound by GABPA, which is consistent with its motif enrichment in these promoters (Fig. 4b). In contrast, much fewer upregulated genes are directly bound by GABPA (Fig. 4b). These results indicate that GABPA directly binds to promoters to activate these genes in E4.5 ICM.

By separating E4.5 ICM genes into early and late ICM genes, we found that GABPA mainly bound to the promoters of early ICM genes (for example, *Tdgf1*, *Etv5*, *Dppa4* and *Uhrf1*), but not late ICM genes (Fig. 4c,d). In addition to the promoter, GABPA also exhibited distal binding, which were putative enhancer regions. We calculated the distance between the TSS of ICM/TE genes and their nearest distal GABPA peaks and found that the TSS of the ICM genes was closer to the distal GABPA peaks than that of the TE genes (Fig. 4e), indicating a potential role of these distal GABPA bindings in regulating ICM genes. Indeed, we detected binding of GABPA to the known super-enhancer of ICM gene *Sox2*^29^ (Extended Data Fig. 5c).

To investigate whether GABPA would affect chromatin accessibility and/or promoter/enhancer activity, we performed ATAC–seq as well as H3K27ac CUT&RUN in E4.5 ICM with or without GABPA degradation (Extended Data Fig. 5d). We found that GABPA degradation resulted in a widespread decrease of H3K27ac with little effect on chromatin accessibility (Fig. 4f,g and Extended Data Fig. 5e). This result indicates that, while GABPA is not responsible for chromatin opening, it is important for the transcriptional activity of the bound genes. Previous reports of the interaction between GABPA and p300 (refs. ^30,31^) in combination with the fact that GABPA degradation results in a decrease in H3K27ac level suggest that GABPA may activate its targets by recruiting the acetyltransferase p300.

### GABPA regulates a common sets of EPI genes in E4.5 ICM and naive ES cells

**Fig. 5:**
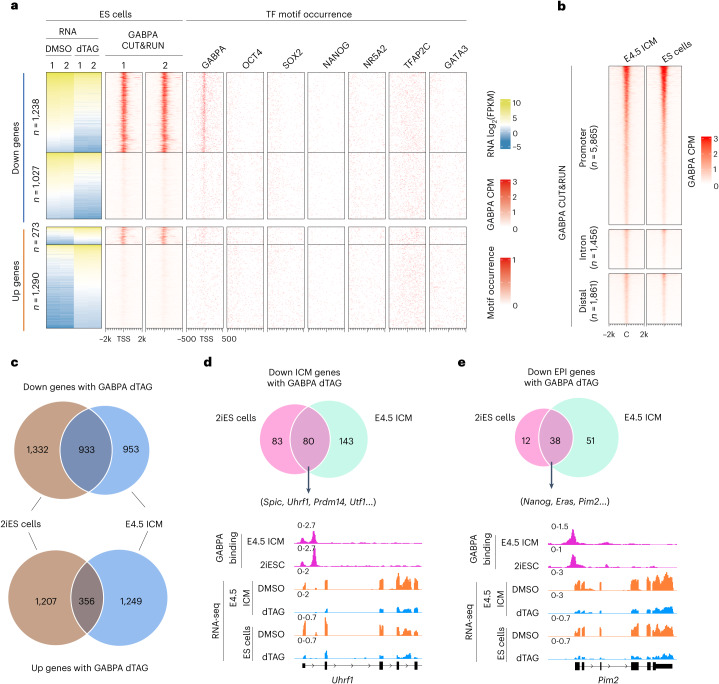
GABPA regulates a common set of EPI genes in E4.5 ICM and ES cells. **a**, Heatmaps showing the DEGs in 2iES cells after dTAG13 treatment for 24 h, and the GABPA binding GABPA motif occurrence around the TSS of the indicated gene groups. **b**, Heatmaps showing similar GABPA binding peaks in E4.5 ICM and 2iES cells. Two replicates were merged for GABPA binding analysis in E4.5 ICM and ES cells. **c**–**e**, A comparison of the DEGs (**c**), downregulated ICM genes (**d**) and downregulated EPI genes (**e**) between E4.5 ICM and 2iES cells after dTAG13 treatment. RNA-seq and GABPA CUT&RUN analysis were performed using two independent biological replicates. **b**, **d** and **e** show the pooled results of the two biological replicates in each condition.

**Extended Data Fig. 6:**
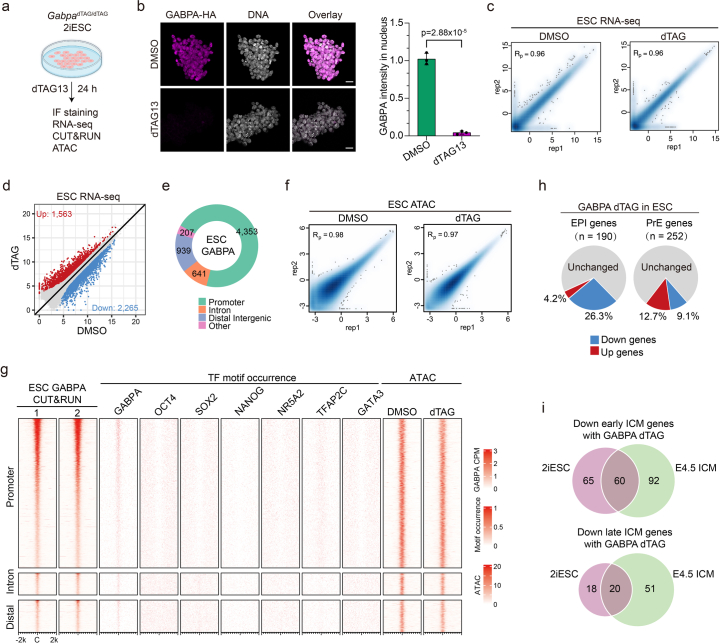
GABPA degradation in ESCs. (**a**) Diagram illustrating the GABPA degradation by dTAG13 treatment in *Gabpa*^dTAG/dTAG^ ESCs. (**b**) Immunostaining confirming GABPA degradation after dTAG13 treatment in ESCs (n = 3). Scale bar, 20 μm. Data are presented as mean values ± SD. (**c**) Correlations of replicates RNA-seq in ESCs. R_p_: Pearson correlation. (**d**) Dot plot showing the DEGs in ESCs after GABPA degradation. (**e**) Genomic distribution of GABPA binding peaks in ESCs. (**f**) Correlation of replicates for ATAC-seq with or without dTAG13 treatment in ESCs. R_p_: Pearson correlation. (**g**) Heatmaps showing the GABPA binding profiles (n = 2 independent biological replicates) at promoter, intron and distal regions in ESCs, and the TFs motif occurrence and ATAC signals (Pooled results from two independent biological replicates were showed) at the corresponding regions. C: peak center. (**h**) Percentage of down-regulated EPI and PrE genes after dTAG13 treatment in ESCs. (**i**) Comparisons of the down-regulated early and late ICM genes upon dTAG13 treatment in ESCs and E4.5 ICM.

Since E4.5 ICM is composed of EPI and PrE cells, it is technically challenging to obtain pure EPI cells to evaluate the role of GABPA in regulating EPI gene expression. To overcome this challenge, we used 2iES cells as they are believed to resemble the E4.5 EPI^32^. Since *Gabp*a KO affects ES cell survival^33^, we used the *Gabpa*^dTAG/dTAG^ ES cells treated with dTAG13 to evaluate the acute effect of GABPA degradation (Extended Data Fig. 6a). GABPA degradation was confirmed by western blot analysis (Fig. 1e) and immunostaining (Extended Data Fig. 6b). RNA-seq revealed 2,265 downregulated genes and 1,563 upregulated genes in response to GABPA degradation for 24 h (Extended Data Fig. 6c,d and Supplementary Table 5). Importantly, GABPA CUT&RUN analysis revealed that 1,238 (54.7%) of the downregulated genes have GABPA promoter binding (Fig. 5a and Extended Data Fig. 6e). Similar to that in E4.5 ICM, GABPA binding motif was enriched in the GABPA peaks and its removal had little effect on ATAC–seq signals (Extended Data Fig. 6f,g). In contrast, the upregulated genes have much less GABPA direct promoter binding (Fig. 5a). Comparative analysis confirmed that GABPA binding in E4.5 ICM and ES cells was highly similar (Fig. 5b), suggesting that GABPA may regulate the same set of genes in ES cells and E4.5 ICM. Indeed, analysis of the differentially expressed genes (DEGs) in E4.5 ICM and ES cells revealed significant overlap with 933 commonly downregulated and 356 commonly upregulated genes in response to GABPA degradation (Fig. 5c). These results indicate that GABPA regulates a similar set of genes in E4.5 ICM and 2iES cells.

We further found that 26.3% EPI genes were downregulated and 12.7% PrE genes were upregulated by GABPA degradation (Extended Data Fig. 6h), suggesting that GABPA plays an important role in pluripotency regulation in ES cells. Further analysis of the downregulated ICM and EPI genes revealed that 80 ICM genes are in common (for example, *Spic*, *Utf1*, *Prdm14* and *Utf1*) (Fig. 5d), including 60 early ICM genes and 20 late ICM genes (Extended Data Fig. 6i). The analysis also revealed 38 EPI genes in common (for example, *Nanog*, *Eras* and *Pim2*) (Fig. 5e). Collectively, data from E4.5 ICM and 2iES cells support the notion that GABPA plays a crucial role in regulating naive pluripotency both in vivo and in vitro.

### Stepwise pluripotency establishment controlled by TFAP2C/SOX2/GABPA

**Extended Data Fig. 7:**
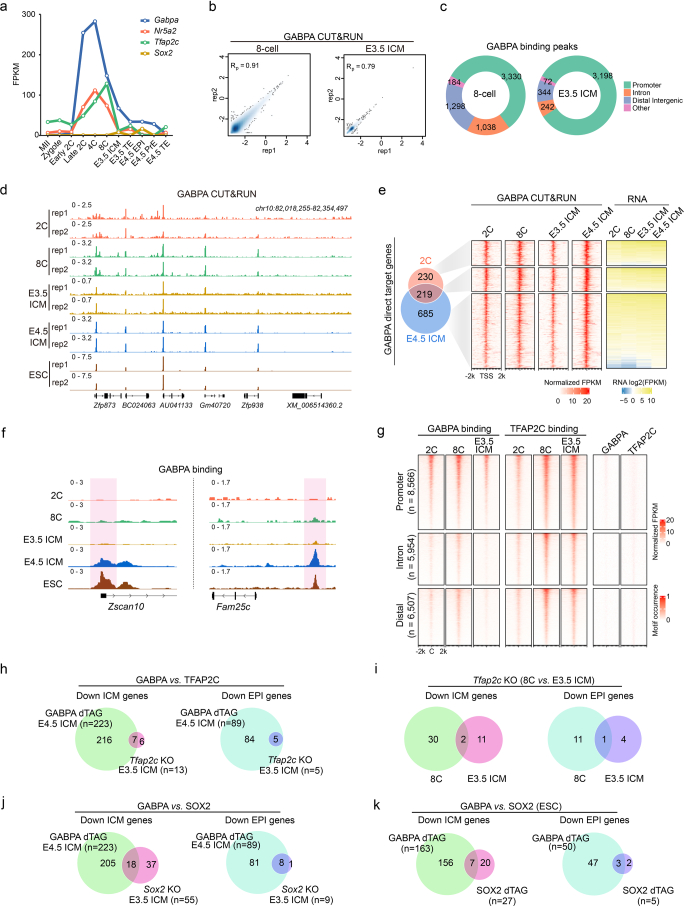
GABPA binding dynamics and comparisons with other pluripotency regulators. (**a**) Expression dynamics of *Gabpa*, *Nr5a2*, *Tfap2c* and *Sox2* during mouse pre-implantation development. (**b**) Correlation of replicates for GABPA CUT&RUN in 8-cell embryos (left) and E3.5 ICM (right). R_p_: Pearson correlation. (**c**) Genomic distribution of GABPA binding peaks in 8-cell embryos (left) and E3.5 ICM (right). (**d**) Genome browser view showing examples of the binding profiles of GABPA in 2-cell, 8-cell, E3.5 ICM, E4.5 ICM and ESCs. (**e**) Comparison of the GABPA direct target genes between 2-cell embryos and E4.5 ICM. (**f**) Examples of genome browser view illustrating the GABPA binding (shaded) dynamics during preimplantation development. (**g**) Heatmap comparison of GABPA and TFAP2C binding profiles at promoter, intron and distal regions in 2-cell, 8-cell and E3.5 ICM. (**h**) Comparisons of GABPA (E4.5 ICM) and TFAP2C (E3.5 ICM) affected ICM genes (left) and EPI genes (right). (**i**) Comparisons of TFAP2C affected ICM genes (left) and EPI genes (right) in 8-cell and E3.5 ICM. (**j**) Comparisons of GABPA (E4.5 ICM) and SOX2 (E3.5 ICM) affected ICM genes (left) and EPI genes (right). (**k**) Comparisons of GABPA and SOX2 affected ICM genes (left) and EPI genes (right) in ESCs. GABPA CUT&RUN and RNA-seq in **e**–**g** showed the pooled results from two biological replicates.

The first and second cell fate specification in mouse embryo occurs at E3.5 and E4.5 with pluripotency gene expression restricted within ICM and EPI, respectively^34–36^. However, the expression of some ICM genes starts as early as the two-cell stage (Fig. 1b). A recent study revealed the role of the TFs NR5A2 and TFAP2C in activating ICM genes at the eight-cell stage^10^. However, the expression of *Nr5a2* and *Tfap2c* is respectively silenced at E3.5 ICM and E4.5 EPI (Extended Data Fig. 7a), indicating that other TFs are responsible for the ICM gene activation at these stages. Our data indicate that GABPA plays such a role for EPI gene activation at E4.5.

**Fig. 6:**
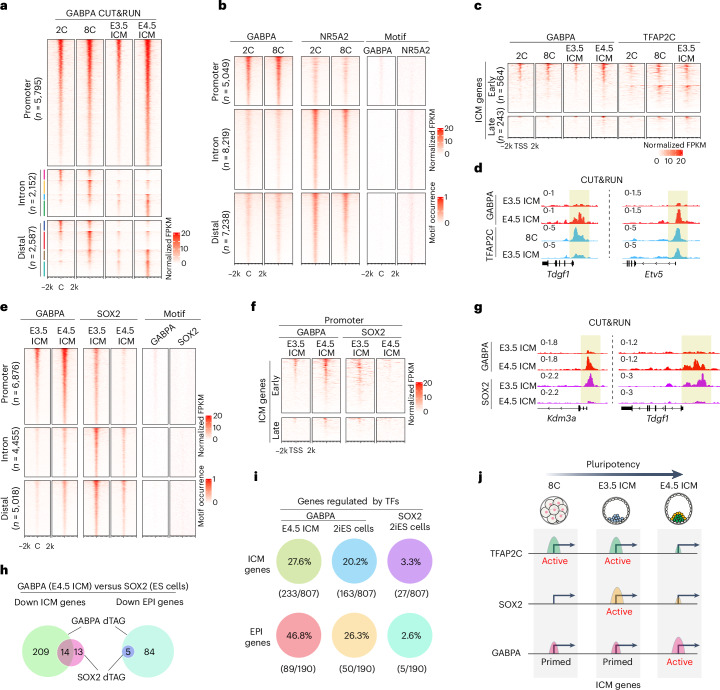
GABPA genomic binding dynamics during pluripotency establishment and its relationship with other pluripotency regulators. **a**, Heatmaps showing the dynamics of GABPA genomic binding profile from two-cell (2C), eight-cell (8C) and E3.5 ICM to E4.5 ICM at promoter, intron and distal regions. C: peak centre. **b**, A comparison of GABPA and NR5A2 binding profiles and motif occurrence at promoter, intron and distal regions at two-cell and eight-cell stages. **c**, GABPA and TFAP2C binding profiles at promoters of early and late ICM genes in two-cell, eight-cell and E3.5 ICM. **d**, The genome browser view of two examples showing GABPA and TFAP2C binding dynamics. Promoter binding regions are shaded. **e**, GABPA and SOX2 binding profiles at promoter, intron and distal regions in E3.5 ICM and E4.5 ICM. **f**, GABPA and SOX2 binding profiles at promoters of early and late ICM genes in E3.5 ICM and E4.5 ICM. **g**, The genome browser view of two examples showing the binding (shaded) of GABPA and SOX2 in E3.5 ICM and E4.5 ICM. **h**, Comparisons of GABPA (E4.5 ICM) and SOX2 (ES cells) affected ICM genes (left) and EPI genes (right) after dTAG13 treatments. **i**, The percentage of ICM and EPI genes regulated by GABPA and SOX2 in E4.5 ICM and ES cells. **j**, A model illustrating that different TFs including TFAP2C, SOX2 and GABPA play major roles at different stages during pluripotency establishment (from the eight-cell to the E4.5 ICM). CUT&RUN analysis in **a**–**g** was performed using two independent biological replicates, and pooled results were used.

To understand how GABPA participates in this process, we generated additional GABPA CUT&RUN dataset in eight-cell and E3.5 ICM (Extended Data Fig. 7b,c). Comparative analysis of GABPA binding profiles at two-cell, eight-cell, E3.5 ICM and E4.5 ICM indicates that strong promoter GABPA binding occurs at the two-cell stage and is maintained through E4.5 ICM (Fig. 6a and Extended Data Fig. 7d), which is consistent with the continuous expression of their target genes (Extended Data Fig. 7e). Weak promoter binding at the two-cell stage became stronger at the E4.5 ICM stage (Fig. 6a). Increased GABPA binding to promoters of ICM genes at E4.5 ICM probably contributes to GABPA’s stage-specific functions (Fig. 6a and Extended Data Fig. 7f).

Next, we analysed the relationship between GABPA and NR5A2 by comparing their binding profiles at the two-cell and eight-cell stages. Interestingly, GABPA tends to bind to promoters, while NR5A2 preferentially binds to putative enhancers (Fig. 6b), suggesting that they have distinct mechanisms in gene regulation. We also compared the binding profiles of GABPA and TFAP2C at two-cell, eight-cell and E3.5 ICM and found that TFAP2C and GABPA co-occupy a portion of ICM genes promoters at eight-cell and E3.5 ICM (Extended Data Fig. 7g), which may explain why GABPA degradation at these stages does not show phenotype. Given that TFAP2C is not expressed in E4.5 EPI (Extended Data Fig. 7a)^9^, while GABPA binding is increased from E3.5 ICM to E4.5 ICM, GABPA may take over TFAP2C’s function on their commonly occupied ICM gene promoters (Fig. 6c,d). To identify the commonly regulated genes taken over by GABPA, we compared the downregulated genes in GABPA-dTAG E4.5 ICM with the downregulated genes in *Tfap2c* maternal-zygotic KO E3.5 ICM and identified that 7 out of the 13 *Tfap2c* KO downregulated ICM genes and all the 5 *Tfap2c* KO downregulated EPI genes showed overlap (Extended Data Fig. 7h). Since *Tfap2c* maternal-zygotic KO at the eight-cell stage showed more downregulated ICM and EPI genes than that in E3.5 ICM (Extended Data Fig. 7i), TFAP2C plays more important role in activating pluripotency genes at the eight-cell than in E3.5 ICM, which is consistent with the observation that *Tfap2c* expression is nearly undetectable at E3.5 ICM (Extended Data Fig. 7a)^9^.

*Sox2* starts to express at E3.5 ICM and plays an important role in regulating pluripotency (Extended Data Fig. 7a)^8^. We thus compared the binding profiles of GABPA and SOX2 in E3.5 ICM and E4.5 ICM. We found a global increase in GABPA binding concomitant with a global decrease in SOX2 binding, especially on promoters from E3.5 ICM to E4.5 ICM (Fig. 6e). Consistently, GABPA binding on ICM gene promoters was enhanced, while SOX2 binding on ICM gene promoters was lost (Fig. 6f,g), indicating the regulation of ICM genes by GABPA was enhanced at E4.5 stage. To explore the possibility that GABPA takes over the role of SOX2 in activating their commonly regulated ICM genes at E4.5, we compared the downregulated genes in GABPA-dTAG E4.5 ICM and *Sox2-*mzKO E3.5 ICM and found that 18 out of the 55 *Sox2-*KO downregulated ICM genes and 8 out of the 9 *Sox2-*KO downregulated EPI genes showed overlap (Extended Data Fig. 7j), supporting the notion that GABPA takes over SOX2’s role on these ICM gene promoters in E4.5 ICM. To gain further support for this notion, we compared the downregulated genes of GABPA-dTAG E4.5 ICM with those of the SOX2-dTAG 2iES cells. Although SOX2 degradation in 2iES cells downregulated only a small number of ICM (27) and EPI (5) genes, all the SOX2 downregulated EPI genes were also downregulated by GABPA and more than half of the SOX2 downregulated ICM genes were also downregulated by GABPA in embryo (Fig. 6h). Three out of the 5 SOX2-dTAG downregulated EPI genes and 7 out of the 27 downregulated ICM genes were also downregulated by GABPA degradation in ES cells (Extended Data Fig. 7k). Collectively, these data support our notion that TFAP2C and SOX2 are responsible for activating pluripotency genes at eight-cell and E3.5 ICM, respectively, while GABPA mainly activates pluripotency genes during E3.5 ICM to E4.5 EPI transition. GABPA takes over the role of TFAP2C and SOX2 at the promoter of pluripotency genes in E4.5 EPI in addition to activating its unique targets.

## Discussion

ZGA and pluripotency acquisition are two of the most important events during pre-implantation development. Understanding the molecular mechanisms underlying these events is not only important in the field of development but also critical for regenerative medicine. Here, we identified and demonstrated that a minor ZGA factor GABPA not only regulates major ZGA but also plays a critical role in pluripotency establishment by activating a large group of pluripotency genes in E4.5 EPI. Despite the observation that GABPA KO downregulates pluripotency factors *Nanog* and *Oct4* in ES cells^33,37^, its role as a key pluripotency regulator was not recognized due to the lack of direct genome binding data. Additionally, its KO lethality phenotype before blastocyst formation^21^ prevented its role in pluripotency in vivo from being addressed using the conventional KO mouse model. In this study, using the dTAG system, we reveal the developmental stage-specific function of GABPA during mouse pre-implantation development. Our data support that GABPA plays a non-dispensable role in ZGA and serves as a master regulator of pluripotency establishment at E4.5 EPI.

The identification of TFs important for mammalian ZGA has been a hot topic in the past several years. Previous studies have been mainly focused on maternal factors that regulate mouse ZGA such as OBOX^26^, NR5A2^28^, DUX^27^, NFYA^38^ and KLF17^39^, while the contributions of zygotic early transcribed genes are neglected, although published work has shown that minor ZGA is required for major ZGA in mice^40^. Here, we provide evidence demonstrating that the minor ZGA factor GABPA regulates a subset of major ZGA genes and pre-implantation development by directly binding to the promoter of 83 major ZGA genes to activate their transcription (Fig. 2g). Our study indicates that minor ZGA gene product can regulate major ZGA by directly binding to and activating major ZGA. Further studies of the other minor ZGA genes are warranted to fully understand the role of minor ZGA genes in regulating major ZGA and pre-implantation development.

Another important finding of this study is that we identify GABPA as a key TF driving the E4.5 EPI specification. We showed that GABPA degradation affects EPI, but not PrE, formation (Fig. 3d–h), which could be explained by GABPA’s role in activating pluripotency genes including *Nanog* and *Sox2* (Fig. 3m). Different from *Nanog* and *Sox2*, which are exclusively expressed in EPI, *Gabpa* is expressed in both EPI and PrE. This expression pattern raises an intriguing question about why GABPA selectively activates EPI genes, but not PrE genes (Fig. 3l,m). One possibility is that binding of GABPA to the pluripotency gene promoters requires another factor that is expressed only in EPI. Alternatively, PrE may express a protein that can mask GABPA binding to promoters. Future studies should confirm or refute these possibilities.

Establishment of pluripotency is a continuous and complex process. Although the expression of pluripotency genes such as *Nanog*, *Sox2* and *Oct4* are critical for maintaining pluripotency^41–44^, their role in pluripotency establishment has not been shown. At eight-cell and E3.5 ICM, TFs such as NR5A2, TFAP2C and SOX2 are believed to modulate the activity of these pluripotency genes^8,9^. They are unlikely to have a major role in EPI specification as they either are not expressed in EPI or they only occupy the promoters of very few pluripotency genes in EPI. Our data indicate that GABPA can fulfil such a role as it regulates 46.8% and 26.3% of EPI genes in E4.5 and 2iES cells, respectively (Fig. 6i). Although the lack of a dTAG mouse model for SOX2 prevented a direct comparison of the role of GABPA and SOX2 in EPI formation, a comparison of dTAG13-mediated degradation of GABPA or SOX2 in 2iES cells showed a clear difference as GABPA degradation downregulated many more ICM genes and EPI genes than did SOX2 degradation (Fig. 6i). Our study, together with previous studies, supports a stepwise model for naive pluripotency establishment (Fig. 6j). At the eight-cell stage, TFAP2C binds to gene promoters and enhancers to active pluripotency genes. At E3.5, with the decrease of TFAP2C expression in ICM, its role in pluripotency gene regulation decreases, while SOX2 starts to function. By E4.5, TFAP2C is no longer expressed in ICM (Extended Data Fig. 7a)^9^, and the binding of SOX2 to the promoters of ICM genes also disappeared, while GABPA now occupies and activates almost half of the pluripotency genes in EPI. Our study thus not only identifies GABPA as an important TF for ZGA but also demonstrates its crucial role as a master regulator of naive pluripotency in E4.5 embryos.

## Methods

### Animals

All experiments were conducted in accordance with the National Institute of Health Guide for Care and Use of Laboratory Animals and approved by the Institutional Animal Care and Use Committee of Boston Children’s Hospital and Harvard Medical School (protocol number IS00000270-6). All mice were kept under specific pathogen-free conditions within an environment controlled for temperature (20–22 °C) and humidity (40–70%), and were subjected to a 12 h light/dark cycle. The generation of *Gabpa*-dTAG knock-in mice was as described previously with some modifications^23^. Briefly, two-cell embryos (20 hpf) were injected with *Gabpa* donor DNA (30 ng μl^−1^), Cas9 mRNA (100 ng μl^−1^) and sgRNA (single guide RNA) (50 ng μl^−1^) using a Piezo impact-driven micromanipulator (Primer Tech). Then, two-cell embryos were incubated in KSOM (potassium simplex optimized medium) for 2 h before transfer into oviducts of pseudo-pregnant Institute of Cancer Research (ICR) strain mothers (Charles River). F0 chimera mice were backcrossed with wild-type C57BL/6J mice for at least two generations. Genotyping was performed using mouse tail lysed in lysis buffer (50 mM Tris–HCl, 0.5% Triton and 400 μg ml^−1^ Proteinase K) at 55 °C overnight. For F0 and F1 mice genotyping, the primers outside the homology arm are used. For genotyping of F2 and beyond, the inner primers are used. The primers are listed in Supplementary Table 6.

Droplet digital PCR (ddPCR) was used for detecting the copy number of *Gabpa*-dTAG knock-in alleles in F1 mice. Briefly, 250 ng purified DNA templates were digested by incubation with Haelll Enzyme (NEB) at 37 °C for 1 h and then inactivated at 80 °C for 5 min. A final 30 ng of DNA was used as the templates for PCR. *Fkbp* was used for knock-in detecting, and *mRPP30* was used as a control. The primers are included in Supplementary Table 6. Only the mice with a single *Fkbp* copy (Supplementary Table 7) were used for further mating.

### mES cell culture and establishment of *Gabpa*^dTAG/dTAG^ cell line

The laboratory-maintained ES-E14 cells^45^ were cultured on 0.1% gelatin-coated plates with 2i/LIF (MEKi/GSK3i/leukemia inhibitory factor) condition. Cells were grown in Dulbecco’s modified Eagle medium (Gibco, 11960069), supplemented with 15% foetal bovine serum (Sigma-Aldrich, F6178), 2 mM GlutaMAX (Gibco, 35050061), 1 mM sodium pyruvate (Gibco, 11360), 1× MEM NEAA (minimum essential medium nonessential amino acids) (Gibco, 11140050), 0.084 mM 2-mercaptoethanol (Gibco, 21985023), 1 mM sodium pyruvate (Gibco, 11360070), 100 U ml^−1^ penicillin–streptomycin (Gibco, 15140122), 1,000 IU ml^−1^ LIF (Millipore, ESG1107), 0.5 μM PD0325901 (Tocris, 4192) and 3 μM CHIR99021 (Tocris, 4423).

To establish *Gabpa*^dTAG/dTAG^ cell line, *Gabpa*-HAL-FKBP^F36V^-2xHA-HAR and px330 were transfected into mouse (m)ES cells with Lipofectamine 2000 (Thermo, 11668030). Twenty-four hours later, cells were selected with puromycin (Gibco, A1113803) for another 48 h. Then, cells were cultured in puromycin-free medium for 1 week. Single clones were picked for genotyping and further analysis.

### In vitro fertilization and embryo culture

Female mice (7–8 weeks) were superovulated through an initial injection of 7.5 IU pregnant mare serum gonadotropin (BioVendor, RP1782725000), followed 48 h later with a 7.5 IU injection of human chorionic gonadotropin (Sigma, C1063). Oocyte–cumulus complexes (OCCs) were collected 14 h post human chorionic gonadotropin injection. Sperm was collected from the cauda epididymis of adult male mice (8–12 weeks) 1 h before OCC collection. The sperm suspension was capacitated for 1 h in 200 μl human tubal fluid (HTF) medium (Millipore, MR-070-D). Subsequently, OCCs were exposed to spermatozoa for a 6 h incubation. The time when sperm was added to OCCs was considered as 0 hpf. Two-nuclear zygotes were cultured in the KSOM medium (Millipore, MR-106-D) under a humidified atmosphere of 5% CO_2_ at 37 °C for further development.

### Western blot

A total of 1.5 × 10^6^ cells were lysed in 100 µl RIPA lysis buffer and incubated on ice for 30 min. Ninety microlitres of supernatant was mixed with 12.5 µl 5× loading buffer, and heated at 98 °C for 15 min. Samples were run on NuPAGE 4–12% gel (Invitrogen, NP0322BOX) and transferred onto polyvinylidene fluoride transfer membrane. Primary antibodies used included anti-GABPA (1:4,000, Proteintech), anti-HA (1:1,000, CST) and anti-β-actin (1:5,000, CST, #4967). Secondary antibodies used included goat anti-Rabbit IgG (H + L) superclonal secondary antibody-HRP (Thermo Scientific, A27036, 1:2,000) and goat anti-mouse IgG (H + L) secondary antibody-HRP (Thermo Fisher Scientific, 31430, 1:2,000). Protein bands were detected with an enhanced chemiluminescence (ECL) kit (Thermo Fisher Scientific, 32209) and imaged by Tanon 4600SF Imaging System (Tanon).

### Immunostaining and confocal microscope

Embryos were fixed with 4% paraformaldehyde/0.5% Triton for 20 min, followed by three times of washing with phosphate-buffered saline/0.1% Triton, then blocked in phosphate-buffered saline/1% bovine serum albumin/0.01% Triton for 1 h. Embryos were incubated overnight at 4 °C with primary antibodies: anti-GABPA (1:200, Proteintech, 21542-1-AP, lot no. 00018047), anti-HA (1:200, CST, 2367S), anti-GATA4 (1:200, R&D Systems, MAB2606-SP), anti-NANOG (1:200, Abcam, ab80892) and anti-CDX2 (1:500, R&D Systems, AF3665-SP). Secondary antibodies used included donkey anti-goat IgG (H + L) secondary antibody, Alexa Fluor 647 (Thermo Scientific, A-21447, 1:500), donkey anti-rabbit IgG (H + L) secondary antibody, Alexa Fluor 488 (Thermo Scientific, A-21206, 1:500) and Donkey anti-mouse IgG Secondary Antibody, Alexa Fluor 568 (Fisher Scientific, A10037, 1:500). After three times of washing, the embryos were incubated with secondary antibodies at room temperature (RT). DNA was stained with 10 μg ml^−1^ Hoechst 33342 (Sigma). The confocal microscope (Zeiss, LSM800) was used for fluorescence detection.

### dTAG13 treatment

dTAG13 (Tocris, 6605) was reconstituted in DMSO to a 5 mM stock. For *Gabpa*^dTAG/dTAG^ mES cell treatment, dTAG13 was diluted in mES cell culture medium to 0.5 μM. For *Gabpa*^dTAG/dTAG^ embryo treatment, dTAG13 was diluted in KSOM to 1 μM. Embryos were washed with KSOM with dTAG13 at least three times, then cultured in KSOM with dTAG13 for further development.

### CUT&RUN, ATAC and RNA-seq library preparation and sequencing

For mES cell CUT&RUN with more than 10,000 cells were resuspended in 50 μl washing buffer (20 mM HEPES pH 7.5, 150 mM NaCl, 0.5 mM spermidine and 1× protease inhibitor) with activated Concanavalin A magnetic beads (Polysciences, 86057-3) for 10 min at RT, then samples were incubated with anti-GABPA (1:40, Proteintech, 21542-1-AP, lot#00018047; note that this is the only lot that worked in CUT&RUN in our hands) overnight at 4 °C. For low-input mES cells and embryo CUT&RUN, some modifications were made. Briefly, mES cells, zona-free embryos or isolated ICM were resuspended in 50 μl washing buffer with activated Concanavalin A magnetic beads for 10 min at RT, then samples were fixed with 1% formaldehyde for 1 min. After three washes with washing buffer, samples were incubated with anti-GABPA overnight at 4 °C. Samples were incubated with 2.8 ng μl^−1^ pA-MNase (home-made) for 2 h at 4 °C. Subsequently, samples were incubated with 200 μl pre-cooled 0.5 μM CaCl_2_ for 20 min at 4 °C and quenched by adding 23 μl 10× stop buffer (1,700 mM NaCl, 20 mM EGTA, 100 mM EDTA, 0.02% digitonin, 250 µg ml^−1^ glycogen and 250 µg ml^−1^ RNase A). DNA fragments were released by incubation at 37 °C for 15 min. For both fixed and unfixed cells, 2.5 μl 10% SDS and 2.5 μl 20 mg ml^−1^ Proteinase K (Thermo Fisher) was added and incubated at 55 °C for at least 1 h for reverse crosslinking. DNA was extracted by phenol–chloroform followed by ethanol precipitation. The subsequent procedure was the same as described above. Sequencing libraries were prepared with NEBNext Ultra II DNA library preparation kit for Illumina (New England Biolabs, E7645S).

ATAC–seq was performed as previously described with some modifications^46^. Briefly, ES cells and isolated ICM were digested with adapter-loaded Tn5 for 15 min at 37 °C, and stopped by stop buffer (100 mM Tris pH 8.0, 100 mM NaCl, 40 µg ml^−1^ Proteinase K and 0.4% SDS) and incubated overnight at 55 °C. Five microlitres of 25% Tween-20 was added to quench SDS. Sequencing libraries were prepared with NEBNext High-Fidelity 2× PCR Master Mix (NEB, M0541S).

For RNA-seq, fresh ES cells or embryos were collected. Reverse transcription and complementary DNA amplification were performed with SMART-Seq v4 Ultra Low Input RNA Kit (Clontech, 634890), followed by cDNA fragmentation, adaptor ligation and amplification using Nextera XT DNA Sample Preparation Kit (Illumina, FC-131-1024).

All libraries were sequenced by NextSeq 550 system (Illumina) with paired-ended 75 bp reads (Supplementary Table 8).

### Immunosurgery

ICMs were isolated as previously described^47^. Briefly, blastocysts at E3.5 or E4.5 stages were collected by removing the zona pellucida with Acidic Tyrode’s solution (Millipore). Embryos were then treated with anti-mouse serum antibody (Sigma-Aldrich, M5774-2ML, 1:5 dilution in KSOM) for 30 min at 37 °C. After washing three times with KSOM, embryos were treated with guinea pig complement (Millipore, 1:5 dilution in KSOM) for another 20 min at 37 °C. Then, the TE cells were removed by a glass pipette (the inner diameter is around 40–50 μm). E4.5 ICM refers to the mixture of EPI and PrE cells after removing TE cells with immunosurgery.

### RNA-seq data analysis

The raw sequencing reads were trimmed with Trimmomatic^48^ (v0.39) to remove sequencing adaptors. Then, the reads were mapped to GRCm38 genome using STAR^49^ (v2.7.8a). Gene expression levels were quantified with RSEM^50^ (v1.3.1). To identify differentially expression genes, the DESeq2^51^ (v1.32.0) package in R was used. The significantly differentially expressed genes were called with an adjusted *P*-value cut-off of 0.05, fold change cut-off of 2 and mean FPKM (fragments per kilobase of transcript per million mapped reads) cut-off of 1. GO enrichment was performed using R package clusterProfiler^52^. Gene Set Enrichment Analysis (GSEA) was performed using R clusterProfiler^52^ and enrichplot.

### CUT&RUN data analysis

The raw reads were trimmed with Trimmomatic^48^ (v0.39) to remove sequencing adaptors, then mapped to GRCm38 reference genome using bowtie2^53^ (v2.4.2). PCR duplicates were removed with Picard MarkDuplicates (v2.23.4). Reads with mapping quality less than 30 were removed. The mapped reads were further filtered to retain only proper paired reads with fragment length between 10 and 120. Peaks were called with MACS2^54^ (v2.2.7.1). Reproducible peaks were generated with the irreproducible discovery rate (IDR) framework^55^ using two replicates, with IDR threshold of 0.05. For E4.5 ICM and ES cells, we further filtered the peaks to keep the ones with *q* value ≤10^−30^ to remove weak peaks. The signal tracks were generated with deeptools^56^ bamCoverage (v3.5.1) with bin size of 1 and normalized by CPM (counts per million). For *z*-score-normalized signal tracks, we first used bamCoverage with bin size of 100 to generate FPKM signals, then used a customized script to calculate the *z* score of each bin. For late two-cell and eight-cell GABPA ultralow-input CUT&RUN data, we noticed low mapping rates of the raw data. Further examination of the unmapped reads suggested they were environmental DNA from human, bacteria, vectors and so on, and due to the ultralow-input cells and small number of GABPA binding regions, the ratio of mapped reads from GABPA-bound DNA versus unmapped reads arising from environmental DNA was low. However, this would not affect the identification of GABPA peaks, since (1) the discarded reads were unmappable to mouse reference genome and (2) these peaks disappeared upon GABPA degradation.

The peaks were annotated with R package ChIPseeker^57^. Peaks within −1,000 to +500 around the TSS were considered as promoter peaks.

The heatmaps of binding profiles were calculated with deeptools^56^ computeMatrix (v3.5.1) using bigwig signal tracks as input and bin size of 10, and visualized in R with packages profileplyr and EnrichedHeatmap^58^.

### ATAC–seq data analysis

ATAC–seq data were analysed with the ENCODE^59^ ATAC–seq pipeline with default parameters (v2.1.2, https://github.com/ENCODE-DCC/atac-seq-pipeline).

### Motif enrichment analysis

Motif enrichment analysis was performed with HOMER^60^ (v4.11) findMotifsGenome.pl with mm10 reference and parameter size 200, using peaks file as input.

### Motif occurrence analysis

Motif occurrence analysis was performed with HOMER^60^ (v4.11) annotatePeaks.pl with parameters ‘mm10 -size -2000,2000 -hist 20 -ghist’ for peak regions and parameters ‘mm10 -size -500,500 -hist 20 -ghist’ for regions around gene TSSs. The motif files were download from JASPAR database^61^ and manually converted to HOMER motif format. We used the log odds detection threshold of 6.0 for all TFs we analysed. The motif occurrence matrix was visualized in R with package EnrichedHeatmap^58^.

The JASPAR motif IDs for the TFs we analysed were as follows: GABPA, MA0062.2; OBOX, PH0121.1; DUX, MA0611.1; NFYA, MA0060.1; NR5A2, MA0505.1; OCT4, MA1115.1; SOX2, MA0143.1; NANOG, MA2339.1; TFAP2C, MA0524.2; GATA3, MA0037.4.

### Identification of ICM/TE genes

ICM/TE genes were identified using bulk RNA-seq data of E3.5 ICM and E3.5 TE from Zhang et al.^18^. The DESeq2^51^ (v1.32.0) package in R was used to find the DEGs between ICM and TE, with an adjusted *P*-value cut-off of 0.05 and fold change cut-off of 2. Genes with eight-cell FPKM ≥1 were defined as early ICM/TE genes, while genes with eight-cell FPKM <1 were defined as late ICM/TE genes.

### Identification of EPI/PrE genes

EPI/PrE genes were identified using single-cell RNA-seq data of E4.5 embryos from Mohammed et al.^62^. The R package Seurat^63^ (v5.0.1) function FindMarkers was used to identify the DEGs between E4.5 EPI and E4.5 PrE, with an adjusted *P*-value cut-off of 0.05, fold change cut-off of 4 and cell expression percentage cut-off of 0.8. Genes with expression in at least 50% E3.5 single cells were considered as early EPI/PrE genes, while the others were considered as late EPI/PrE genes.

### Statistics and reproducibility

Student’s *t*-tests for graph analysis were performed with Microsoft Excel (2016). Individual data points are shown as dots in the figure panels involving the Student’s *t*-test. Data distribution was assumed to be normal, but this was not formally tested. For the immunofluorescence and western blot experiments, at least three independent repetitions were performed with consistent results, and representative data were presented. *P* values < 0.05 were considered statistically significant. No statistical methods were used to pre-determine sample sizes, but our sample sizes are similar to or greater than those reported in previous publications^8^. No data were excluded from the analyses. The experiments were not randomized. Data collection and analysis were not performed blind to the conditions of the experiments.

### Reporting summary

Further information on research design is available in the Nature Portfolio Reporting Summary linked to this article.

## Online content

Any methods, additional references, Nature Portfolio reporting summaries, source data, extended data, supplementary information, acknowledgements, peer review information; details of author contributions and competing interests; and statements of data and code availability are available at 10.1038/s41556-024-01554-0.

## Supplementary information

Reporting Summary

Supplementary Table 1 Gene lists used in this study.

Supplementary Table 2 DEGs of late two-cell stage treated with GABPA dTAG13 versus that treated with DMSO.

Supplementary Table 3 DEGs of morula treated with GABPA dTAG13 versus that treated with DMSO.

Supplementary Table 4 DEGs of E4.5 ICM treated with GABPA dTAG13 versus that treated with DMSO.

Supplementary Table 5 DEGs of ES cell treated with GABPA dTAG13 versus that treated with DMSO.

Supplementary Table 6 Oligos used in this study.

Supplementary Table 7 ddPCR validate GabpadTAG knock-in copy number.

Supplementary Table 8 Summary of the sequenced libraries in this study.

## Source data

Source Data Fig. 1 Unprocessed western blots for Fig.1.

Source Data Fig. 2 Statistical source data for Figs. 1–6 and Extended Data Figs. 1–7.

## Data Availability

Sequencing data that support the findings of this study have been deposited in the Gene Expression Omnibus (GEO) under accession code GSE263171. Public data used in this study: RNA-seq of mouse MII oocyte to eight-cell^17^: GSE71434. RNA-seq of mouse E3.5 ICM and TE^18^: GSE76505. RNA-seq of mouse E4.5 TE^9^: GSE216256. Single-cell RNA-seq of mouse E4.5 EPI and PrE^64^: GSE159030. Single-cell RNA-seq of mouse early embryos^19^: GSE45719. Single-cell RNA-seq of mouse E3.5 and E4.5 embryos^62^: GSE100597. ATAC–seq of mouse early embryos^16^: GSE66390. NR5A2 binding in mouse embryos^10^: GSE229740. TFAP2C binding in mouse embryos^9^: GSE216256. SOX2 binding in mouse embryos^8^: GSE203194. Source data are provided with this paper. All other data supporting the findings of this study are available from the corresponding author on reasonable request.
