# Supplementary material for: The transcription factor GABPA is a master regulator of naive pluripotency
Source: Nat Cell Biol. 2025 Jan 2;27(1):48–58. doi: 10.1038/s41556-024-01554-0 (PMC11735382; doi:10.1038/s41556-024-01554-0)
Supplement: Supplementary file 1 — Reporting Summary [file 41556_2024_1554_MOESM1_ESM.pdf]

nature portfolio

Corresponding author(s): Yi Zhang

Last updated by author(s): Sep 17, 2024

Reporting Summary

Nature Portfolio wishes to improve the reproducibility of the work that we publish. This form provides structure for consistency and transparency in reporting. For further information on Nature Portfolio policies, see our Editorial Policies and the Editorial Policy Checklist.

Statistics

For all statistical analyses, confirm that the following items are present in the figure legend, table legend, main text, or Methods section.

n/a	Confirmed	
☐	☒	The exact sample size (*n*) for each experimental group/condition, given as a discrete number and unit of measurement
☐	☒	A statement on whether measurements were taken from distinct samples or whether the same sample was measured repeatedly
☐	☒	The statistical test(s) used AND whether they are one- or two-sided<br>*Only common tests should be described solely by name; describe more complex techniques in the Methods section.*
☒	☐	A description of all covariates tested
☐	☒	A description of any assumptions or corrections, such as tests of normality and adjustment for multiple comparisons
☐	☒	A full description of the statistical parameters including central tendency (e.g. means) or other basic estimates (e.g. regression coefficient) AND variation (e.g. standard deviation) or associated estimates of uncertainty (e.g. confidence intervals)
☐	☒	For null hypothesis testing, the test statistic (e.g. *F*, *t*, *r*) with confidence intervals, effect sizes, degrees of freedom and *P* value noted<br>*Give P values as exact values whenever suitable.*
☒	☐	For Bayesian analysis, information on the choice of priors and Markov chain Monte Carlo settings
☒	☐	For hierarchical and complex designs, identification of the appropriate level for tests and full reporting of outcomes
☒	☐	Estimates of effect sizes (e.g. Cohen's *d*, Pearson's *r*), indicating how they were calculated

Our web collection on statistics for biologists contains articles on many of the points above.

Software and code

Policy information about availability of computer code

Data collection	The data collection methods for RNA-seq, CUT&RUN and ATAC-seq were described in Methods section. The immunostaining images were collected from the ZEISS ZEN Microscopy software (v2.3). The quantification of the immunostaining images was calculated with ImageJ (v1.50i).

Data analysis	The data analysis methods were described in Methods section. The software used in data analysis and the corresponding version were provided below:<br><br>Trimmomatic: v0.39<br>STAR: v2.7.8a<br>RSEM: v1.3.1<br>DESeq2: v1.32.0<br>clusterProfiler: v4.10.0<br>bowtie2: v2.4.2<br>Picard: v2.23.4<br>MACS2: v2.2.7.1<br>deeptools: v3.5.1<br>ChIPseeker: v1.38.0<br>profileplyr: v1.18.0<br>EnrichedHeatmap: v1.32.0<br>HOMER: v4.11<br>R: 4.3.2

For manuscripts utilizing custom algorithms or software that are central to the research but not yet described in published literature, software must be made available to editors and reviewers. We strongly encourage code deposition in a community repository (e.g. GitHub). See the Nature Portfolio guidelines for submitting code & software for further information.

Data

Policy information about availability of data

All manuscripts must include a data availability statement. This statement should provide the following information, where applicable:

- Accession codes, unique identifiers, or web links for publicly available datasets
- A description of any restrictions on data availability
- For clinical datasets or third party data, please ensure that the statement adheres to our policy

Sequencing data that support the findings of this study have been deposited in the Gene Expression Omnibus (GEO) under accession code GSE263171. Public data use in this study: RNA-seq of mouse MII oocyte to 8-cell: GSE71434. RNA-seq of mouse E3.5 ICM and TE: GSE76505. RNA-seq of mouse E4.5 TE: GSE216256. scRNA-seq of mouse E4.5 EPI and PrE: GSE159030. scRNA-seq of mouse early embryos: GSE45719. scRNA-seq of mouse E3.5 and E4.5 embryos: GSE100597. ATAC-seq of mouse early embryos: GSE66390. NR5A2 binding in mouse embryos: GSE229740. TFAP2C binding in mouse embryos: GSE216256. SOX2 binding in mouse embryos: GSE203194. The GRCm38 reference genome was downloaded from http://ftp.ensembl.org/pub/release-98/fasta/mus_musculus/dna/Mus_musculus.GRCm38.dna.primary_assembly.fa.gz . Source data are provided with this study. All other data supporting the findings of this study are available from the corresponding author on reasonable request.

Research involving human participants, their data, or biological material

Policy information about studies with human participants or human data. See also policy information about sex, gender (identity/presentation), and sexual orientation and race, ethnicity and racism.

Reporting on sex and gender	N/A
Reporting on race, ethnicity, or other socially relevant groupings	N/A
Population characteristics	N/A
Recruitment	N/A
Ethics oversight	N/A

Note that full information on the approval of the study protocol must also be provided in the manuscript.

Field-specific reporting

Please select the one below that is the best fit for your research. If you are not sure, read the appropriate sections before making your selection.

☒ Life sciences ☐ Behavioural & social sciences ☐ Ecological, evolutionary & environmental sciences

For a reference copy of the document with all sections, see nature.com/documents/nr-reporting-summary-flat.pdf

Life sciences study design

All studies must disclose on these points even when the disclosure is negative.

Sample size	No statistical methods were used to predetermine sample size. Sample size and number of replicates were chosen based on other studies (PMID: 33821005, 38096290).
Data exclusions	No data was excluded from the analysis.
Replication	All the RNA-seq, ATAC, CUT&RUN were repeated twice, and other experiments were repeated at least three times. And all of them were succeed.
Randomization	Strains and conditions that were directly compared were typically cultured together. Microscopy image acquisition was performed randomly. All samples were allocated randomly into experimental groups. Further randomization was not applicable.
Blinding	Blinding was not involved in the experiments. This should not affect the interpretation of the data. All the data collection are done by one person and were therefore not blinded.

Reporting for specific materials, systems and methods

We require information from authors about some types of materials, experimental systems and methods used in many studies. Here, indicate whether each material, system or method listed is relevant to your study. If you are not sure if a list item applies to your research, read the appropriate section before selecting a response.

Materials & experimental systems

n/a	Involved in the study
☐	☒ Antibodies
☐	☒ Eukaryotic cell lines
☒	☐ Palaeontology and archaeology
☐	☒ Animals and other organisms
☒	☐ Clinical data
☒	☐ Dual use research of concern
☒	☐ Plants

Methods

n/a	Involved in the study
☐	☒ ChIP-seq
☒	☐ Flow cytometry
☒	☐ MRI-based neuroimaging

Antibodies

Antibodies used

anti-GABPA (1:200, Proteintech, 21542-1-AP, Lot#00018047), anti-HA (1:200, CST, 2367S), anti-GATA4 (1:200, R&D Systems, MAB2606-SP, Monoclonal Mouse IgG2B Clone # 532020), anti-NANOG (1:200, Abcam, ab80892) and anti-CDX2 (1:500, R&D Systems, AF3665-SP). anti-β-actin (1:5000, CST, #4967). Second antibodies used included Goat anti-Rabbit IgG (H+L) Superclonal™ Secondary Antibody-HRP (Thermo Scientific, A27036, 1:2000) and Goat anti-Mouse IgG (H+L) Secondary Antibody-HRP (Thermo Fisher Scientific, 31430, 1:2000). Donkey anti-Goat IgG (H+L) Secondary Antibody, Alexa Fluor 647 (Thermo Scientific, A-21447, 1:500), Donkey anti Rabbit IgG (H+L) Secondary Antibody, Alexa Fluor 488 (Thermo Scientific, A-21206, 1:500) and Donkey anti Mouse IgG Secondary Antibody, Alexa Fluor 568 (Fisher Scientific, A10037, 1:500).

Validation

All the antibodies are from commercial source and have been validated by the vendors and their validation data are available on the published papers and manufacturers' website (Abcam, R&D Systems, Cell Signaling and Proteintech).

anti-GABPA:
western blot and IF were verified by the published paper (PMID: 23684612) and antibody website: https://www.ptglab.com/products/NRF2-Antibody-21542-1-AP.htm. CUT &RUN was verified by us. With dTAG treatment, all the peaks disappear.

anti-HA:
western blot and IF were verified by the published paper (PMID: 38878777) and antibody website: https://www.cellsignal.com/products/primary-antibodies/ha-tag-6e2-mouse-mab/2367

anti-GATA4:
IF were verified by the published paper (PMID: 32104112) and antibody website:https://www.rndsystems.com/products/human-gata-4-antibody-532020_mab2606

anti-NANOG:
IF were verified by the published paper (PMID: 36525967) and antibody website. https://www.abcam.com/products/primary-antibodies/nanog-antibody-ab80892.html. The website clarify that this antibody is suitable for: ICC, ICC/IF, IHC-P, Flow Cyt, IHC - Wholemount, WB, and reacts with: Mouse.

anti-cdx2:
IF were verified by the published paper (PMID: 34861147) and antibody website. https://www.rndsystems.com/products/human-cdx2-antibody_af3665

anti-beta-actin:

WB were verified by the published paper (PMID: 39155876) and antibody website. The website clarify that this antibody is suitable for: WB, and the species reactivity: Human, Mouse, Rat, Hamster, Monkey, Mink, D. melanogaster, Zebrafish, Bovine

Eukaryotic cell lines

Policy information about cell lines and Sex and Gender in Research

Cell line source(s)	The ES-E14 cell line was previous used in this lab. The E14 cell line was kindly provided by the laboratory of B. Koller. https://pubmed.ncbi.nlm.nih.gov/31209294/
Authentication	The transcriptome of our mESCs is highly similar to that of a published ES-E14 dataset on enocde project (Pearson correlation, r=0.92). https://pubmed.ncbi.nlm.nih.gov/31209294/
Mycoplasma contamination	The ESCs are mycoplasma free.
Commonly misidentified lines (See ICLAC register)	None of the cell lines used in this study is listed in the database of commonly misidentified cell lines maintained by ICLAC

Animals and other research organisms

Policy information about studies involving animals; ARRIVE guidelines recommended for reporting animal research, and Sex and Gender in Research

Laboratory animals	Generation of knock-in mice was as described previously with some modification 23. Briefly, 2-cell embryo (20 hpf) were injected with Gabpa donor DNA (30 ng/μl), Cas9 mRNA (100 ng/μl) and sgRNA (50 ng/μl) using a Piezo impact-driven micromanipulator (Primer Tech, Ibaraki, Japan). Then 2-cell embryos were incubated in KSOM for 2 hrs before transferred into oviducts of pseudo-pregnant ICR strain mothers (Charles River). F0 chimera mice was backcross with wild-type C57BL/6J mice for at least two generation. Female mice (7-8 weeks) and adult male mice (8-12 weeks) were used for IVF experiments
Wild animals	No wild animals were used in the study
Reporting on sex	Female mice were used for oocyte collection, and male mice were used for sperm collection
Field-collected samples	This study did not involve field-collected samples.
Ethics oversight	All animal experiments were performed in accordance with the protocols of the Institutional Animal Care and Use Committee at Harvard Medical School. All mice were kept under specific pathogen-free conditions within an environment controlled for temperature (20-22°C) and humidity (40-70%), and were subjected to a 12-hour light/dark cycle.

Note that full information on the approval of the study protocol must also be provided in the manuscript.

Plants

Seed stocks	N/A
Novel plant genotypes	N/A
Authentication	N/A

ChIP-seq

Data deposition

☒ Confirm that both raw and final processed data have been deposited in a public database such as GEO.

☒ Confirm that you have deposited or provided access to graph files (e.g. BED files) for the called peaks.

Data access links *May remain private before publication.*	All data generated in this study have been deposited to the NCBI Gene Expression Omnibus (GEO) with accession number GSE263171.
Files in database submission	GSM8187096 GABPA_CUTrun_2cell_rep1

Files in database submission

GSM8187097	GABPA_CUTrun_2cell_rep2
GSM8187098	GABPA_CUTrun_8cell_rep1
GSM8187099	GABPA_CUTrun_8cell_rep2
GSM8187100	GABPA_CUTrun_E35_ICM_rep1
GSM8187101	GABPA_CUTrun_E35_ICM_rep2
GSM8187102	GABPA_CUTrun_E45_ICM_rep1
GSM8187103	GABPA_CUTrun_E45_ICM_rep2
GSM8187104	GABPA_CUTrun_ESC_rep1
GSM8187105	GABPA_CUTrun_ESC_rep2
GSM8187106	GABPA_CUTrun_ESC_500
GSM8187107	GABPA_dTAG_CUTrun_2cell_rep1
GSM8187108	GABPA_dTAG_CUTrun_2cell_rep2
GSM8187109	GABPA_dTAG_CUTrun_E45_ICM_rep1
GSM8187110	GABPA_dTAG_CUTrun_E45_ICM_rep2
GSM8187111	ATAC_E45_ICM_Gabpa_DMSO_rep1
GSM8187112	ATAC_E45_ICM_Gabpa_DMSO_rep2
GSM8187113	ATAC_E45_ICM_Gabpa_dTAG_rep1
GSM8187114	ATAC_E45_ICM_Gabpa_dTAG_rep2
GSM8187115	ATAC_ESC_Gabpa_DMSO_rep1
GSM8187116	ATAC_ESC_Gabpa_DMSO_rep2
GSM8187117	ATAC_ESC_Gabpa_dTAG_24h_rep1
GSM8187118	ATAC_ESC_Gabpa_dTAG_24h_rep2
GSM8187119	H3K27ac_E45_ICM_Gabpa_DMSO_rep1
GSM8187120	H3K27ac_E45_ICM_Gabpa_DMSO_rep2
GSM8187121	H3K27ac_E45_ICM_Gabpa_dTAG_rep1
GSM8187122	H3K27ac_E45_ICM_Gabpa_dTAG_rep2
GSM8187123	RNA_L2C_Gabpa_DMSO_rep1
GSM8187124	RNA_L2C_Gabpa_DMSO_rep2
GSM8187125	RNA_L2C_Gabpa_DMSO_rep3
GSM8187126	RNA_L2C_Gabpa_dTAG_rep1
GSM8187127	RNA_L2C_Gabpa_dTAG_rep2
GSM8187128	RNA_L2C_Gabpa_dTAG_rep3
GSM8187129	RNA_morula_Gabpa_DMSO_rep1
GSM8187130	RNA_morula_Gabpa_DMSO_rep2
GSM8187131	RNA_morula_Gabpa_dTAG_rep1
GSM8187132	RNA_morula_Gabpa_dTAG_rep2
GSM8187133	RNA_E45_ICM_Gabpa_DMSO_rep1
GSM8187134	RNA_E45_ICM_Gabpa_DMSO_rep2
GSM8187135	RNA_E45_ICM_Gabpa_dTAG_rep1
GSM8187136	RNA_E45_ICM_Gabpa_dTAG_rep2
GSM8187137	RNA_ESC_Gabpa_DMSO_rep1
GSM8187138	RNA_ESC_Gabpa_DMSO_rep2
GSM8187139	RNA_ESC_Gabpa_dTAG_24h_rep1
GSM8187140	RNA_ESC_Gabpa_dTAG_24h_rep2

Genome browser session
(e.g. UCSC)

no longer applicable

Methodology

Replicates

Each experiment was repeated twice.

Sequencing depth

Each CUT&RUN library was sequenced for at least 25 million read pairs with 2 × 75bp length. The sequencing depth for each library was provided in Supplementary Table 8.

Antibodies

anti-GABPA (1:200, Proteintech, 21542-1-AP, Lot#00018047)

Peak calling parameters

macs2 callpeak -t {input.bam} -f BAMPE -B --SPMR -p 0.01 -g mm --keep-dup all --scale-to large

Data quality

The CUT&RUN data quality was evaluated using the Pearson correlation between two biological replicates. Reproducible peaks were generated with the IDR framework using two replicates, with IDR threshold of 0.05.

Software

The raw reads were trimmed with Trimmomatic (v0.39) to remove sequencing adaptors then mapped to GRCm38 reference genome using bowtie2 (v2.4.2). PCR duplicates were removed with Picard MarkDuplicates (v2.23.4). Reads with mapping quality less than 30 were removed. The mapped reads were further filtered to only retain proper paired reads with fragment length between 10 and 120. Peaks were called with MACS2 (v2.2.7.1).
