# Supplementary figures and images for: The transcription factor GABPA is a master regulator of naive pluripotency
Source: Nat Cell Biol. 2025 Jan 2;27(1):48–58. doi: 10.1038/s41556-024-01554-0 (PMC11735382; doi:10.1038/s41556-024-01554-0)

**Fig.1e**

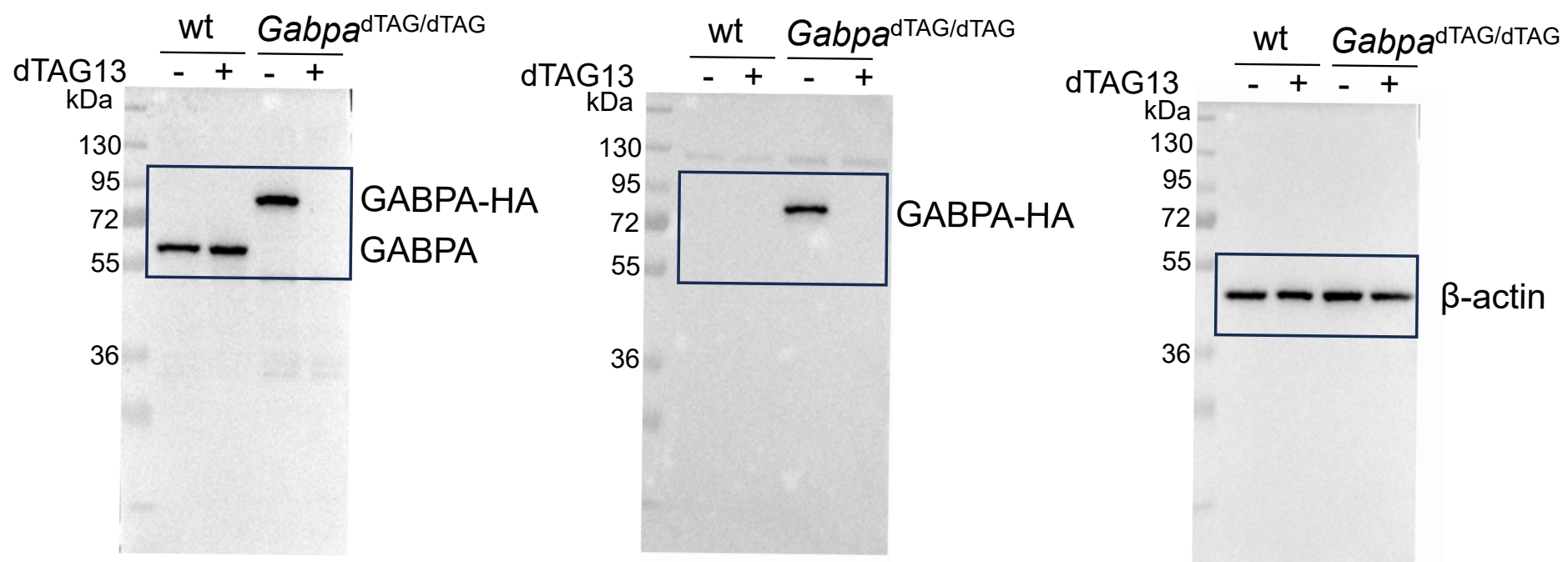

Supplement: Supplementary file 10 — Unprocessed western blots for Fig.1. [file 41556_2024_1554_MOESM10_ESM.pdf]
